# RNA reference materials with defined viral RNA loads of SARS-CoV-2—A useful tool towards a better PCR assay harmonization

**DOI:** 10.1371/journal.pone.0262656

**Published:** 2022-01-20

**Authors:** Laura Vierbaum, Nathalie Wojtalewicz, Hans-Peter Grunert, Vanessa Lindig, Ulf Duehring, Christian Drosten, Victor Corman, Daniela Niemeyer, Sandra Ciesek, Holger F. Rabenau, Annemarie Berger, Martin Obermeier, Andreas Nitsche, Janine Michel, Martin Mielke, Jim Huggett, Denise O’Sullivan, Eloise Busby, Simon Cowen, Peter M. Vallone, Megan H. Cleveland, Samreen Falak, Andreas Kummrow, Thomas Keller, Ingo Schellenberg, Heinz Zeichhardt, Martin Kammel

**Affiliations:** 1 INSTAND e.V., Society for Promoting Quality Assurance in Medical Laboratories, Duesseldorf, North Rhine-Westphalia, Germany; 2 GBD Gesellschaft fuer Biotechnologische Diagnostik mbH, Berlin, Germany; 3 IQVD GmbH, Institut fuer Qualitaetssicherung in der Virusdiagnostik, Berlin, Germany; 4 Institute of Virology, Charité - University Medicine Berlin; National Consultant Laboratory for Coronaviruses; German Centre for Infection Research, Berlin, Germany; 5 Institute for Medical Virology, University Hospital, Goethe University Frankfurt, Frankfurt, Hesse, Germany; 6 German Centre for Infection Research, External partner site Frankfurt, Hesse, Germany; 7 Fraunhofer Institute for Molecular Biology and Applied Ecology, Branch Translational Medicine and Pharmacology, Frankfurt, Hesse, Germany; 8 Medizinisches Infektiologiezentrum Berlin, Germany; 9 Robert Koch-Institute, Centre for Biological Threats and Special Pathogens, Berlin, Germany; 10 Robert Koch-Institute, Department for Infectious Diseases, Berlin, Germany; 11 National Measurement Laboratory, LGC, Teddington, Middlesex, United Kingdom; 12 Faculty of Health & Medical Science, School of Biosciences & Medicine, University of Surrey, Guildford, United Kingdom; 13 Materials Measurement Laboratory, Biomolecular Measurement Division, NIST, National Institute of Standards and Technology, Applied Genetics Group, Gaithersburg, Massachusetts, United States of America; 14 Physikalisch-Technische Bundesanstalt, Berlin, Germany; 15 ACOMED statistik, Leipzig, Saxony, Germany; 16 Institute of Bioanalytical Sciences, Center of Life Sciences, Anhalt University of Applied Sciences, Bernburg, Saxony-Anhalt, Germany; Instituto Butantan, BRAZIL

## Abstract

SARS-CoV-2, the cause of COVID-19, requires reliable diagnostic methods to track the circulation of this virus. Following the development of RT-qPCR methods to meet this diagnostic need in January 2020, it became clear from interlaboratory studies that the reported Ct values obtained for the different laboratories showed high variability. Despite this the Ct values were explored as a quantitative cut off to aid clinical decisions based on viral load. Consequently, there was a need to introduce standards to support estimation of SARS-CoV-2 viral load in diagnostic specimens. In a collaborative study, INSTAND established two reference materials (RMs) containing heat-inactivated SARS-CoV-2 with SARS-CoV-2 RNA loads of ~10^7^ copies/mL (RM 1) and ~10^6^ copies/mL (RM 2), respectively. Quantification was performed by RT-qPCR using synthetic SARS-CoV-2 RNA standards and digital PCR. Between November 2020 and February 2021, German laboratories were invited to use the two RMs to anchor their Ct values measured in routine diagnostic specimens, with the Ct values of the two RMs. A total of 305 laboratories in Germany were supplied with RM 1 and RM 2. The laboratories were requested to report their measured Ct values together with details on the PCR method they used to INSTAND. This resultant 1,109 data sets were differentiated by test system and targeted gene region. Our findings demonstrate that an indispensable prerequisite for linking Ct values to SARS-CoV-2 viral loads is that they are treated as being unique to an individual laboratory. For this reason, clinical guidance based on viral loads should not cite Ct values. The RMs described were a suitable tool to determine the specific laboratory Ct for a given viral load. Furthermore, as Ct values can also vary between runs when using the same instrument, such RMs could be used as run controls to ensure reproducibility of the quantitative measurements.

## 1. Introduction

After the outbreak and global spread of the novel coronavirus disease (COVID-19), caused by the severe acute respiratory syndrome coronavirus 2 (SARS-CoV-2), the situation was officially designated as a pandemic by WHO on March 11, 2020 [1]. To track and thereby control the spread of SARS-CoV-2, methods predominantly utilizing reverse transcription quantitative polymerase chain reaction (RT-qPCR) were deployed to identify the virus and isolate infected individuals in order to interrupt the chains of transmission [2]. As early as January 2020, the first method for detecting SARS-CoV-2 by RT-qPCR was published by an international consortium [3,4]. RT-qPCR allows quantitative estimates to be made on the viral RNA load based on the threshold cycle Ct or other measures defining the quantification cycle Cq [5,6]. As no reference material (RM) for detecting SARS-CoV-2 was available during the first months of the pandemic, gaps in interlaboratory comparability were to be expected. Published data on SARS-CoV-2 RNA proficiency testing partially confirm a wide dispersion of the Ct values [7–14] with laboratories differing by over a 1000-fold for a given Ct/Cq value [14].

However, the reliability of individual RT-qPCR test systems, as well as a good comparability of interlaboratory test results, is crucial for interpreting results and for making appropriate clinical decisions e.g., for estimating the infectivity of a patient for developing criteria for discharging patients from isolation. Data from the literature suggests that the probability of virus cultivation (especially from diagnostic samples taken after symptom onset) is low for diagnostic samples with a viral load below ~10^6^ to ~10^7^ copies/mL (conservatively estimated at about 20%) [12,15–19]. This implies viral RNA quantity could be used as a surrogate to guide patient stratification in terms of risk of transmission or as for criteria for discharging patients from isolation. Furthermore, some groups have suggested using viral quantitative cut offs, using Ct values as units of measure, for this purpose [20–23]. However, the interlaboratory variation outlined above suggests Ct values alone may not be a reliable measure to guide patient stratification.

In order to assess the above-mentioned threshold range of ~10^6^ to ~10^7^ copies/mL, two quantitative RMs were developed (for definition of reference materials see Vocabulary International of Metrology (VIM) and ISO 17511 [24,25]): RM 1 with ~10^7^ copies/mL and RM 2 with ~10^6^ copies/mL. The project was a cooperation between the Robert Koch Institute (RKI), the National Consultant Laboratory for Coronaviruses at the Institute for Virology of the Charité—University Medicine Berlin, INSTAND as a Reference Institution of the German Medical Association (Bundesaerztekammer) for external quality assurance in medical laboratories, as well as members of the Joint Diagnostic Commission of the German Association for the Control of Viral Diseases (DVV) and the Society for Virology (GfV). During the project, three National Metrology Institutes (NMIs) confirmed the viral RNA load by RT digital PCR (RT-dPCR), a calibration free measurement technique [25]. With the aid of these samples, laboratories were able to correlate their procedure-dependent Ct values from diagnostic material to the corresponding Ct values of the samples with an assigned viral RNA load.

As quantitative diagnostic considerations in the genome detection of SARS-CoV-2 have become increasingly required, we investigated whether calibration of interlaboratory Ct values could improve harmonization and therefore patient stratification. Therefore, shortly before the establishment of the WHO International Standard for SARS-CoV-2 RNA (NIBSC code 20/146), the RMs developed in this interdisciplinary project were intended to serve as complementary tools for individual laboratories concerned with the test system- and gene-dependent interpretation of their results. In this study we analyzed 1,109 results of 305 participating German laboratories for these SARS-CoV-2 RMs in relation to gene region and test system. Furthermore, we highlighted the potential of using such RMs to improve the accuracy of molecular tools and provide a more dynamic testing environment to assist our efforts to support informed decisions regarding the SARS-CoV-2 pandemic.

## 2. Materials and methods

### 2.1 Cells and virus

SARS-CoV-2 (strain: BetaCoV/Munich/ChVir984/2020, GISAID: EPI_ISL_406862) was used for the preparation of the quantitative RM 1 and RM 2. The virus, provided by the National Consultant Laboratory for Coronaviruses at Charité—University Medicine Berlin, Institute of Virology, Berlin, Germany, was propagated under BSL-3 conditions in Vero E6 cells (ATCC CRL-1586) which were maintained in a 5% CO_2_ atmosphere at 37 °C in Dulbecco’s Modified Eagle’s Medium, supplemented with 10% fetal bovine serum, 1% non-essential amino acids 100x concentrate and 1% sodium pyruvate 100 mM. Infection of Vero E6 cells was carried out with a passage one virus stock and an MOI of 0.05 PFU/cell. The supernatant of the infected cell cultures was collected three days after infection and heat inactivated (4 h, 60 °C). Inactivation of the virus was proven by two blind passages. The number of plaque-forming units (PFU) in the cell culture supernatant was reduced from 4.6 x 10^5^ PFU/mL to 0 PFU/mL by this heat inactivation.

### 2.2 Quantitative pre-characterization of SARS-CoV-2 in the cell culture supernatant

The SARS-CoV-2 RNA load in the cell culture supernatant described in Section 2.1 was determined by RT-dPCR measurements in the course of the INSTAND EQA Scheme (340) for Virus Genome Detection of Coronaviruses incl. SARS-CoV-2 in June/July 2020 [14]. The SARS-CoV-2 positive sample 340066 of this EQA scheme, containing 1: 5,000,000 diluted cell culture supernatant, was selected for SARS-CoV-2 RNA load quantification by RT-dPCR by the three National Metrology Institutes (NMIs): the National Measurement Laboratory (NML at LGC, UK), the National Institute of Standards and Technology (NIST, USA) and the Physikalisch-Technische Bundesanstalt (PTB, Germany). The lyophilized samples were reconstituted in 1.1 mL molecular biology grade water (PCR grade), extracted using the Qiagen QIAamp Viral RNA mini kit, and eluted (see S1 Table for the volumes used by each laboratory). These eluates were analyzed by RT-dPCR on the Bio-Rad QX200 RT-ddPCR platform using the Bio-Rad one-step RT-ddPCR supermix using the CDC N1 assay, CDC N2 assay [26] and China N assay [27]. The results were analyzed by NML using R version 3.6.1 and RStudio version 1.2.5001.

The assigned value for SARS-CoV-2 RNA load for the EQA sample 340066 was 1,570 ± 360 copies/mL at a level of confidence of 95%, and was used to determine the viral RNA load of the inactivated cell culture supernatant to be approximately 7.85 x 10^9^ copies/mL. The raw data of the RT-dPCR analyses are shown in S2 Table.

The same supernatant of the cell culture infected with SARS-CoV-2, as described in Section 2.1 was used to produce the reference materials RM 1 and RM 2.

### 2.3 Preparation and lyophilization of the RMs

RM 1 and RM 2 were prepared by GBD mbH by diluting the SARS-CoV-2 positive cell culture supernatant as follows:

RM 1 (lot 07469)—1:750 to contain a SARS-CoV-2 RNA load of approximately 1 x 10^7^ copies/mL;
RM 2 (lot 07470)—1:7,500 to contain a SARS-CoV-2 RNA load of approximately 1 x 10^6^ copies/mL.

Dilution was performed using cell culture medium (Minimal Essential Medium, PanBioTech, Aidenbach, Germany) supplemented with non-essential amino acids (PanBioTech); HEPES buffer (PanBioTech) and fetal bovine serum (PanBioTech, gamma irradiated; 15% v/v for supplemented cell culture medium).

In total 2,300 vials each of RM 1 and RM 2 (1.1 mL per vial) were aliquoted in screw cap micro tubes (2.0 mL; Sarstedt, Nuermbrecht, Germany). Before lyophilization, primary freezing of the filled micro tubes was performed at -30 °C (4–12 hours) followed by freezing at -70 °C over night.

Process controlled lyophilization was performed in an Epsilon 2-10D LSC freeze dryer (Martin Christ Gefriertrocknungsanlagen GmbH, Osterode, Germany). The gradual lyophilization profile over a period of 72 hours included: (i) a temperature change from -70 °C to 20 °C and (ii) a pressure change from atmospheric pressure to 6 x 10^−2^ bar. At the end of lyophilization, the micro tubes were manually topped with screw caps. The RMs were stored at <-20 °C until they were shipped to the laboratories at ambient temperature.

### 2.4 Analysis of the homogeneity of RM 1 and RM 2 and assignment of quantitative values for SARS-CoV-2 RNA loads

Before distributing RM 1 and RM 2 to the laboratories, 10 to 15 randomly selected sample sets of RM 1 and RM 2 were tested for homogeneity by the following laboratories:

Laboratory 1—National Consultant Laboratory for Coronaviruses, Institute of Virology, Charité—University Medicine Berlin (Germany); Laboratory 2—University Hospital Frankfurt, Institute of Medical Virology (Frankfurt/M., Germany); Laboratory 3—the Robert Koch Institute, Centre for Biological Threats and Special Pathogens (Berlin, Germany); and Laboratory 4—GBD Gesellschaft fuer Biotechnologische Diagnostik mbH (Berlin, Germany).

The PCRs used by Laboratory 1 and Laboratory 3 are described in detail in Section 2.5.1. Laboratory 2 used two fully automated commercial tests, the ‘Cobas SARS-CoV-2 Test’ on a cobas 6800 system (Roche, Basel, Switzerland) and the ‘Alinity m SARS-CoV-2 Assay’ (Abbott, Cologne, Germany). Laboratory 4 used the ‘QIAamp MinElute Virus Spin Kit’ (Qiagen, Hilden, Germany) for extraction and the ‘Allplex 2010-nCoV Assay’ (Seegene, Duesseldorf, Germany) for amplification.

The homogeneity of both reference materials was reflected by prediction intervals (95% probability) ranging between 0.13 and 1.40 Ct values regardless of the test system used for each of the target genes examined by the four laboratories (S1 Fig, S1 Data).

### 2.5 Determination of SARS-CoV-2 RNA loads of RM1 and RM 2

The SARS-CoV-2 RNA loads of RM 1 and RM 2 were determined by two methods.

#### 2.5.1 RT-qPCR applying synthetic SARS-CoV-2 RNA standards

The determination of SARS-CoV-2 RNA loads in both RMs was performed by RT-qPCR using synthetic SARS-CoV-2 RNA standards.

*2*.*5*.*1*.*1 Quantification by the National Consultant Laboratory for Coronaviruses*, *Institute of Virology*, *Charité*—*University Medicine Berlin (Laboratory 1)*. Viral RNA was purified in parallel using two systems: the ‘QIAamp Viral RNA Mini Kit’ (Qiagen, Hilden, Germany) and the ‘MagNA Pure 96 Viral NA Small Volume Kit’ (Roche, Basel, Switzerland) in accordance with the manufacturer’s instructions. Detection and quantitative assessment by RT-qPCR was done using the RdRP (RNA dependent RNA polymerase) and an E gene target. For RT-qPCR, a 25 μl reaction was set up containing 5 μl of purified RNA, and by using the ‘Life Technologies SuperScript^®^ III One-Step’ enzyme mix and a Roche LightCycler^®^ 480 thermocycler as described above [3].

Assessment of viral RNA concentration was done by applying internal calibration curves for each RT-qPCR run based on serially diluted assay-specific photometrically quantified in-vitro transcribed RNA [3].

*2*.*5*.*1*.*2 Quantification by the Robert Koch Institute*, *Centre for Biological Threats and Special Pathogens*, *Berlin (Laboratory 3)*. RNA was extracted using the ‘QIAamp Viral RNA Mini Kit’ (Qiagen, Hilden, Germany) and PCR was performed with the ‘AgPath-ID^™^ One-Step RT-qPCR Reagents Kit’ (Applied Biosystems, Foster City, CA USA) on a CFX96 real-time PCR cycler (Bio-Rad, Hercules, USA) using 5 μl RNA per reaction as described by Michel et al. [28]. For quantification, a 10-fold dilution series of in vitro transcripts provided by WHO (WHO std 1, starting at 3 x 10^6^ copies/reaction) were used to generate a standard curve. Ct values for the E gene were used to calculate RNA loads per reaction and per mL sample.

#### 2.5.2 Reverse transcription digital PCR (RT-dPCR)

The three National Metrology Institutes measured the SARS-CoV-2 RNA loads for both RMs using the calibration free method of RT-dPCR, applying the CDC N2 assay and China N assay, respectively, as described in Section 2.2. See S3 Table for the volumes used by each laboratory and S4 Table for the RT-dPCR data.

### 2.6 Sample distribution

RM 1 and RM 2 were distributed by INSTAND in three shipments to laboratories in Germany, one on November 3 (134 laboratories), the second on November 17 (115 laboratories) and the third on January 15 (130 laboratories). Participants received three vials per RM. The samples had to be reconstituted with 1.1 mL double distilled water (sterile, pyrogen-free, PCR-grade) for 20 min at room temperature.

### 2.7 Measurement of the RMs and reporting of results by the diagnostic laboratories

The laboratories were asked to measure RM 1 and RM 2 in their routinely used RT-qPCRs (for each test and each gene region of SARS-CoV-2 individually) and to correlate the Ct (Cp, Cq) values obtained for each gene region with the known SARS-CoV-2 RNA load of RM 1 and RM 2. After that, the laboratories were asked to report the Ct values they obtained for each sample and each tested gene region back to INSTAND via the RV-Online platform (https://rv-online.instandev.de). Multiple results per sample could be entered, including results from different measurement dates or different test systems. Furthermore, they were asked to provide detailed information, e.g. the date of the analysis, or the test system(s) used for each analysis, including test kit supplier(s) and test kit(s) (S2 Data). Quantitative values in copies per mL or IU per mL could also be reported. However, due to the low number of quantitative values and thus insufficient statistical significance, they were not evaluated for this paper.

### 2.8 Data evaluation and statistics

We evaluated a total of 1,109 data sets from all three shipments provided by the 305 laboratories. The evaluation was carried out on a gene region-specific basis as well as on a combined gene region-specific and test kit-specific basis.

Values that exceeded a Ct value of 50 were excluded from the analysis because they were most likely transcription errors or methodical outliers. Furthermore, sample swaps were excluded from the evaluation so they would not distort the general quality of the data analysis and interpretation (12 exclusions in total).

To get a general impression of the sample stability, the laboratory results were plotted chronologically, starting from the day of the first measurement by the manufacturer. For the main analysis, the Ct differences based on the data sets for both reference samples were calculated and analyzed on a gene region-specific, manufacturer-specific and test kit-specific basis. The confidence intervals for the difference in means were calculated. To characterize the distributions of the median 95% of measured values the +/- 2 standard deviation (SD) ranges were calculated.

In addition, a Passing Bablok fit was performed evaluate the dependency of the individual differences of the Ct values of both RMs on Ct value.

To get an impression of the general performance of the in-house tests, a comparable heterogenous group of fully automated systems was created. The SD for the fully automated tests were generated by combining all results obtained by these tests.

Basic statistical analyses were performed using jmp from SAS Institute (Cary, North Carolina, USA).

### 2.9 Generation of images

The overlay images were generated using the GIMP—GNU Image Manipulation Program 2.10.2.

## 3. Results

### 3.1 Quantification of RM 1 and RM 2

The target values for RNA viral load for RM 1 and RM 2 were determined by RT-qPCR using synthetic RNAs before the reference materials RM 1 and RM 2 were sent to the diagnostic laboratories in Germany to anchor the Ct values obtained for individual samples in routine diagnostic testing. During the project, additional values for RNA viral load were determined for both samples using RT-dPCR.

**Fig 1:**
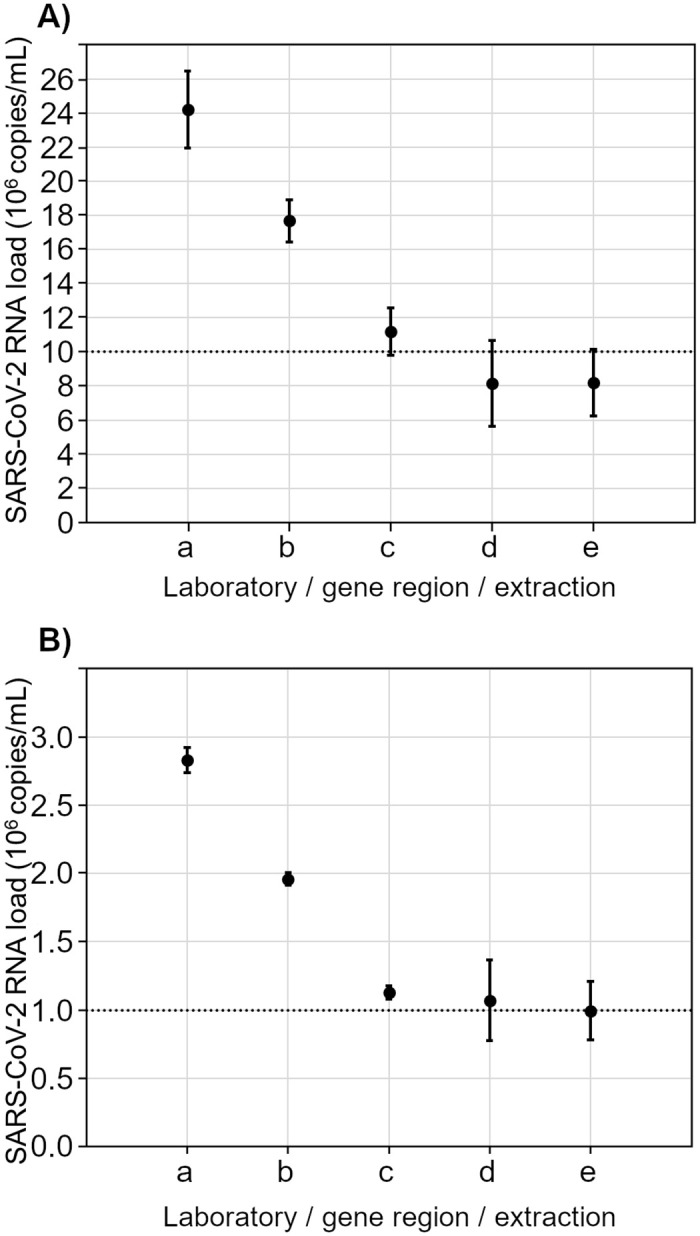
RT-PCR measurement results with 95% confidence intervals on the basis of quantification with synthetic RNA by laboratory 1 and laboratory 3 for RM 1 (A) and RM 2 (B) with details on extraction, amplification and target gene. a = laboratory 1/extraction: Qiagen QIAamp Viral RNA Mini Kit/amplification: in-house/gene region: E gene b = laboratory 1/extraction: Roche MagNA Pure 96 Viral NA Small Volume Kit/amplification: in-house/gene region: E gene c = laboratory 3/extraction: Qiagen QIAamp Viral RNA Mini Kit/amplification: in-house/gene region: E gene d = laboratory 1/extraction: Qiagen QIAamp Viral RNA Mini Kit/amplification: in-house/gene region: RdRP gene e = laboratory 1/extraction: Roche MagNA Pure 96 Viral NA Small Volume Kit/amplification: in-house/gene region: RdRP gene The dotted line in (A) represents the expected SARS-CoV-2 RNA loads of 10^7^ copies/ml for RM 1. The dotted line in (B) represents the expected SARS-CoV-2 RNA loads of 10^6^ copies/ml for RM 2.

**Fig 2:**
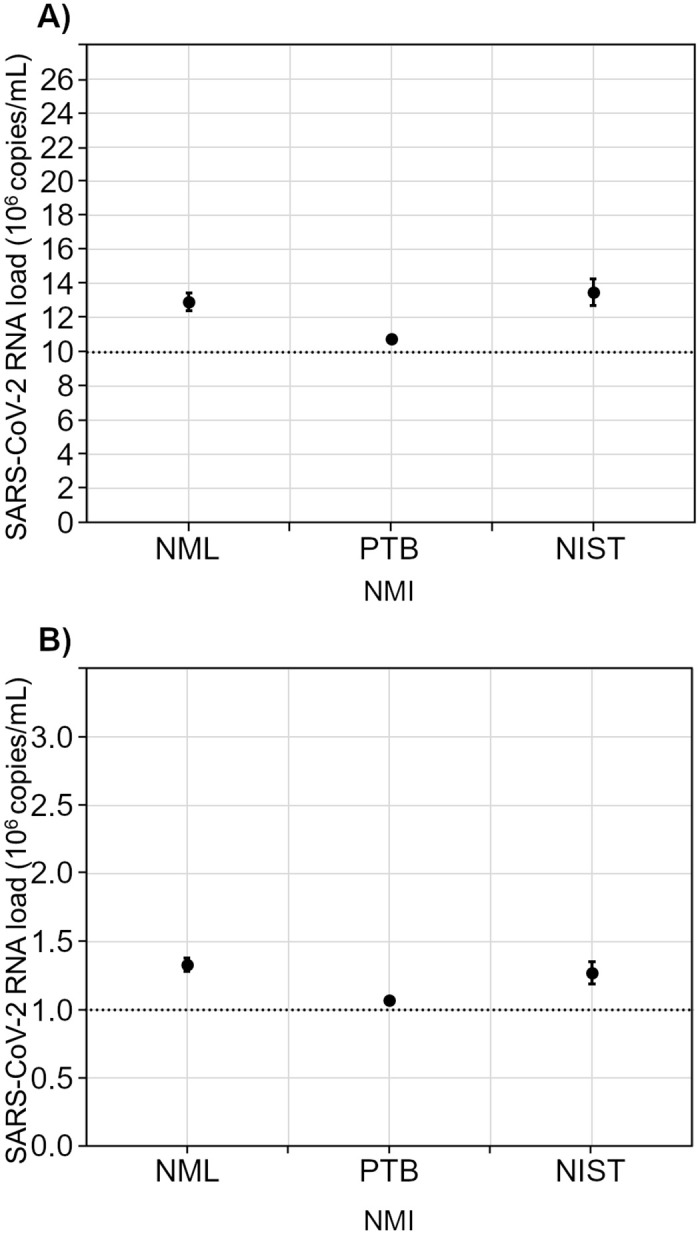
Digital PCR measurement results with 95% confidence intervals from three NMIs for RM 1 (A) and RM 2 (B). The dotted line in (A) represents the expected SARS-CoV-2 RNA loads of 10^7^ copies/mL for RM 1. The dotted line in (B) represents the expected SARS-CoV-2 RNA loads of 10^6^ copies/mL for RM 2.

Depending on the targeted gene region of SARS-CoV-2 and the applied extraction method, the results for quantification by RT-qPCR revealed the anticipated viral RNA loads of between 0.82 and 2.42 x 10^7^ copies/mL for RM 1 and between 0.99 and 2.83 x 10^6^ copies/mL for RM 2 (Fig 1). Analyses by N gene-specific RT-dPCR resulted in SARS-CoV-2 RNA loads for RM 1 of between 1.08 and 1.35 x 10^7^ copies/mL and for RM 2 of between 1.08 and 1.33 x 10^6^ copies/mL (Fig 2).

Taking into account mean and SD calculated from the three NMI specific values, overall mean consensus values (+/- 95%-CI) for the SARS-CoV-2 viral load were assigned to RM 1 and RM 2 (Table 1). A coverage factor of k = 4.3 was used to determine the confidence intervals listed in Table 1 for a level of confidence of 95%.

**Table 1:** Consensus values of the RT-dPCR analyses by three NMIs, reported as the overall mean and its 95%-CI of the three NMI-specific results.

Reference material	Consensus value SARS-CoV-2 RNA load ± expanded uncertainty (copies/mL)
RM 1	(1.24 ± 0.36) x 10^7^
RM 2	(1.23 ± 0.33) x 10^6^

The SARS-CoV-2 RNA load obtained by RT-qPCR and RT-dPCR are in agreement with the aimed for target concentrations and allow RM 1 and RM 2 to be used as reference materials for anchoring the Ct values obtained for individual samples in routine diagnostic testing.

### 3.2 Analysis of data for RM 1 and RM 2 reported by the diagnostic laboratories

RM 1 and RM 2 were distributed to German laboratories in three shipments. The laboratories were able to report their results for both samples with regard to the test systems used by differentiating the respective targeted gene regions. This led to a total of 1,109 data sets for RM 1 and RM 2, respectively, entered by 305 different laboratories over a period of 115 days. 12 results were excluded from the analysis due to inconsistent data.

#### 3.2.1 Development of Ct value distribution over time—Statement on sample stability

**Fig 3:**
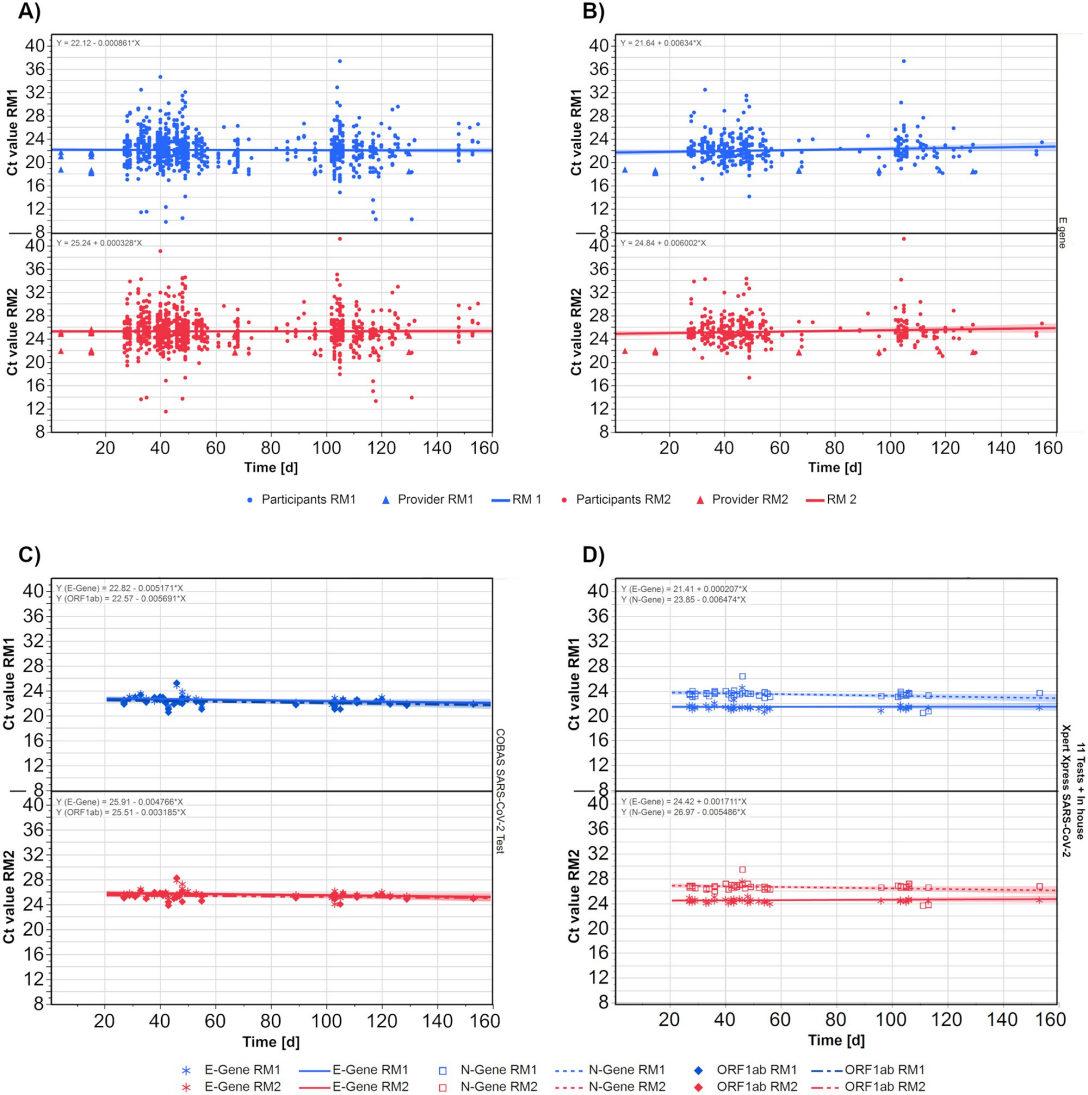
Development of Ct values for both reference samples over time for all results (A), just for E gene results (B), as well as all results the test system Cobas (C) and test system XpertXpress (D) for their respective gene targets. Each symbol represents one measurement. The lines represent the regression lines for the corresponding data set and the formular is displayed in the upper right corner. Dots are participants results and triangles are results from the sample provider (Figure A and B). For Figure C and D no provider results were present, so we used the asterisk for e gene result, the square for N gene results and the diamond for ORF1ab results.

To get an impression of the stability of RM 1 and RM 2, the Ct values of participating laboratories (1,097 data sets) as well as those measured in parallel by the sample provider (63 data sets) were plotted against the elapsed days (Fig 3A). The initial time point was October 8, 2020, when the samples were measured for the first time by the provider.

Despite the scattering of the results, the regression lines for both samples showed an almost horizontal course. This stability in the distribution of Ct values could already be confirmed based on reports for the E gene (Fig 3B) as well as for the other gene regions separately (S2 Fig). To ensure that the regression was not affected by different market leaders of commercial tests, the results for two commonly used assay systems were plotted for their respectively analyzed gene regions (‘COBAS SARS-CoV-2 Assay’ (Fig 3C) and ‘Xpert Xpress SARS-CoV-2’ (Fig 3D)). In both cases, the regression lines showed a stable distribution of values over time. A clear separation between the Ct value distributions for the E gene and the N gene could be observed in the case of the Xpert Xpress assay.

#### 3.2.2 Distribution of Ct values per gene region

**Fig 4:**
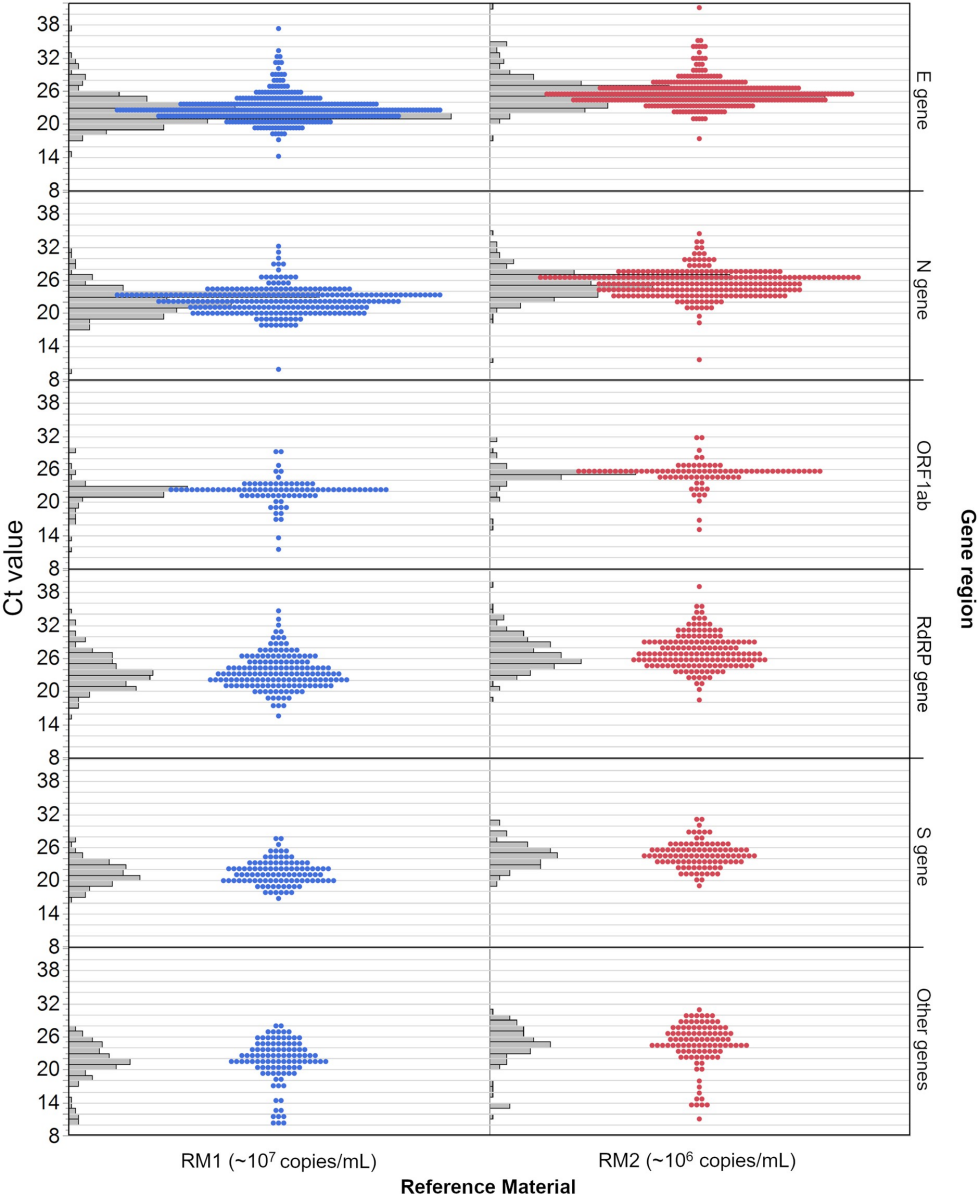
Distribution of all Ct values of participants per gene region. The concentration of RM 1 (blue) is estimated at roughly 10^7^ copies/mL and the concentration of RM 2 (red) is estimated at roughly 10^6^ copies/mL. Grey bars represent the distribution of results, which is also represented by the dots. Each dot indicates one result.

Most results were reported for the E gene (36%), followed by the N gene (24%), RdRP gene (14%), S gene (9%) and ORF1ab (8%) (Table 2). About 9% of the reported results were declared to be ‘other genes’, which were either pooled values of several gene regions, or were not further specified. The distribution of the reported Ct values for the respective gene regions are consistent with a normal distribution, especially for the E gene and the N gene (Fig 4). Although the median Ct values of both samples differed on average by about 3 Ct for each gene region, the distribution of values as well as the +/- 2 SD around the mean showed a clear overlap (Table 2).

**Table 2:** Statistical information (mean, median, 95%-CI of mean) on Ct value distribution per gene region for RM 1 and RM 2.

**(A)**	**RM1**					
**Gene region**	**N**	**Mean**	**95%-CI of mean**	**Median**	**SD**	**+/- 2 SD from mean**
E gene	389	22.2	22.0–22.5	21.9	2.4	17.5–27.0
N gene	259	22.2	21.9–22.5	22.1	2.3	17.5–26.8
ORF1ab	89	21.7	21.2–22.2	22.0	2.4	16.9–26.5
RdRP gene	158	23.5	23.0–24.0	23.2	3.1	17.3–29.7
S gene	100	21.3	20.9–21.7	21.1	2.1	16.9–25.7
Other Gene(s)	101	21.1	20.3–21.8	21.6	3.9	13.4–28.8
**(B)**	**RM2**					
**Gene region**	**N**	**Mean**	**95%-CI of mean**	**Median**	**SD**	**+/- 2 SD from mean**
E gene	389	25.4	25.2–25.7	25.1	2.4	20.6–30.2
N gene	259	25.4	25.1–25.7	25.4	2.4	20.6–30.2
ORF1ab	89	24.8	24.3–25.3	25.1	2.2	20.3–29.3
RdRP gene	158	26.8	26.3–27.2	26.4	3.1	20.5–33.0
S gene	100	24.4	23.9–24.8	24.3	2.2	19.9–28.8
Other Gene(s)	101	24.2	23.4–25.0	24.8	4.1	16.1–32.3

All results that were not specified for either E gene, N gene, ORF1ab, RdRP gene or S gene were collected under ‘other genes’. This also included multi-target tests. The +/- 2 SD ranges were calculated for each gene region and sample.

**Fig 5:**
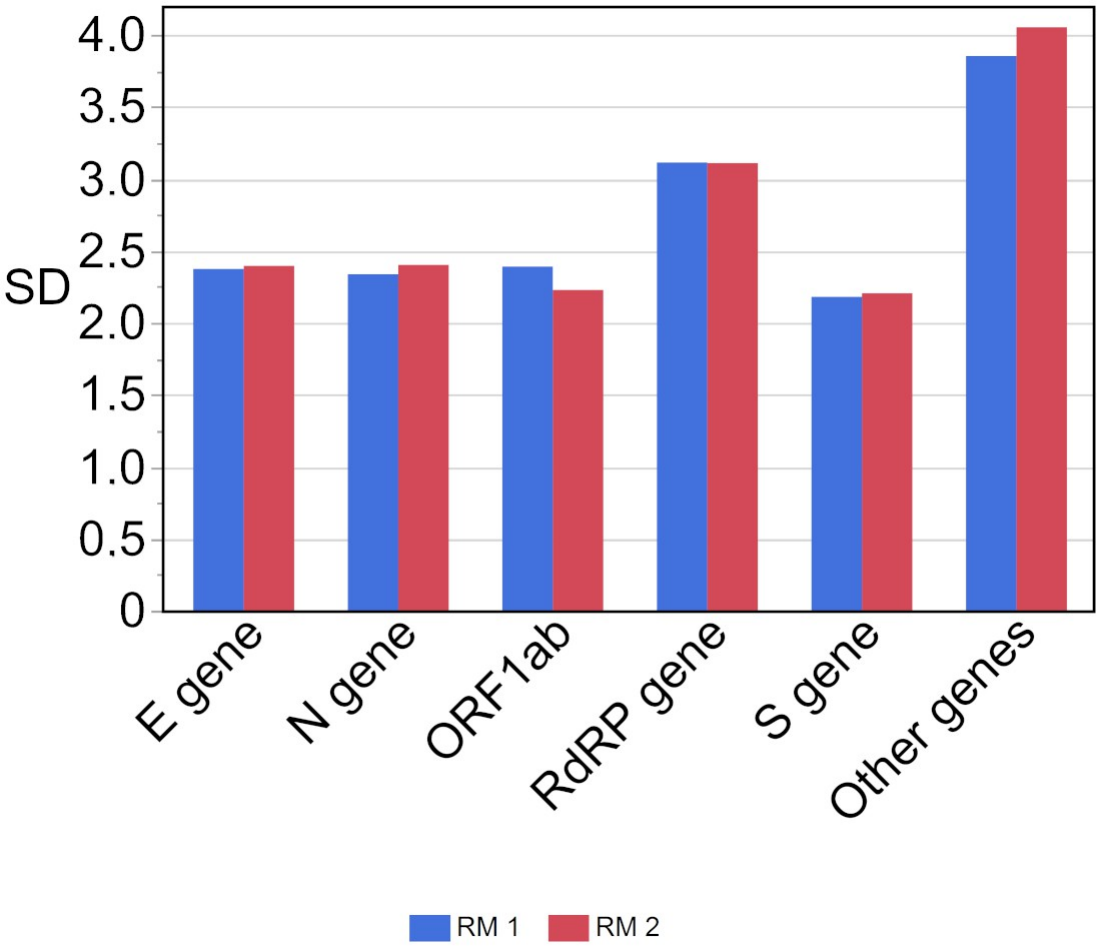
Standard deviation (SD) for the respective Ct value results per gene region. The concentration of RM 1 (blue) is estimated at roughly 10^7^ copies/mL and the concentration of RM 2 (red) is estimated at roughly 10^6^ copies/mL.

The SDs (Fig 5) of the results of both RMs were similar. It was lowest in RM 1 for the S gene (2.181 Ct) and in RM 2 also for the S gene (2.206 Ct). The highest SDs were observed in the ‘other genes’ group (3.854 Ct in RM 1 and 4.054 Ct in RM 2) (Fig 5). The difference in median Ct values for both samples was highest between the S gene and the RdRP gene (~2.1 Ct), whereas it was lowest between the E gene and ORF1ab (0–0.1 Ct). Additional statistical results can be found in S3 Data.

#### 3.2.3 Analysis of test system-based distribution of Ct values

**Fig 6:**
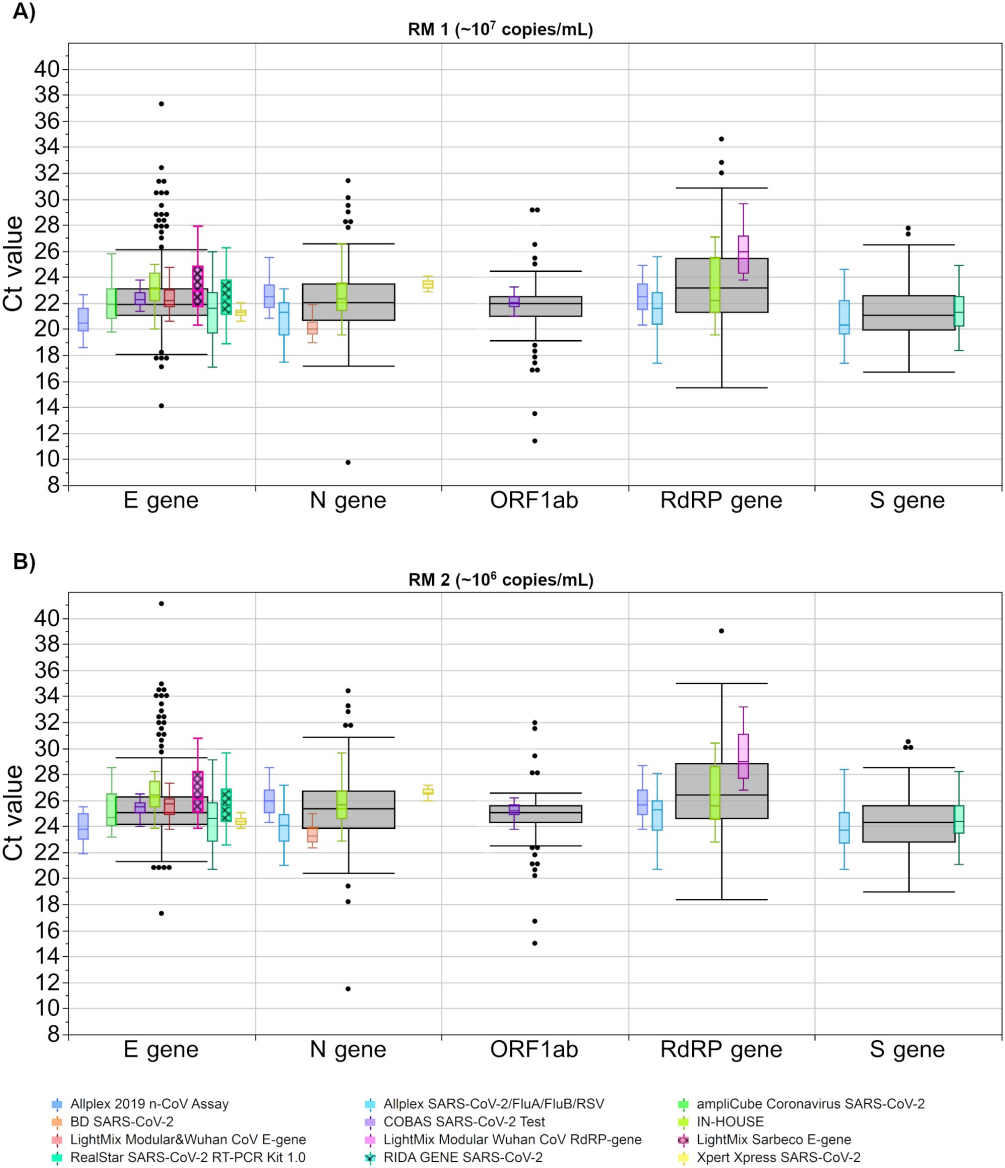
Analysis of Ct values for both samples for different test systems. The concentration of RM 1 (A) is estimated with at 10^7^ copies/mL and the concentration of RM 2 (B) is estimated with at 10^6^ copies/mL. The black boxes display all results for the respective sample, and the distributions of specific manufacturer-based collectives are illustrated as smaller, colored box plots in overlay with the total results. Outlier were excluded from colored boxes. Collectives are shown for eleven test kits. For all boxes, the whiskers stretch from the 1st quartile—1.5*(interquartile range) to the 3rd quartile + 1.5*(interquartile range).

To evaluate the performance of different test systems, we analyzed eleven commonly used assays, including various tests from the same manufacturer, as well as of in-house tests (Fig 6). Fifteen results, classified as ‘other gene regions’, were excluded from this evaluation due to the high heterogeneity of possible targets as well as the low number of values, which would have an insufficient statistical significance.

Median differences of assays with the lowest and the highest Ct values for the corresponding gene regions ranged from ~ 1 Ct for the S gene to ~ 4.4 Ct for the RdRP gene. Test systems from the same manufacturer showed differences ranging from ~ 0.4 Ct to ~ 1.9 Ct depending on the sample and gene region. Interestingly, two tests from Seegene, the ‘Allplex SARS-CoV-2/FluA/FluB/RSV Assay’ and the ‘Allplex 2019 n-CoV Assay’, showed similar Ct values in the case of the RdRP gene. However, for the N gene, the ‘Allplex SARS-CoV-2/FluA/FluB/RSV Assay’ displayed notably lower Ct values than the ‘Allplex 2019 n-CoV Assay’ (up to 1.9 Ct in RM 2). This might be due to the fact that the amplification procedure of the ‘Allplex SARS-CoV-2/FluA/FluB/RSV Assay’ comprises 3 additional cycles upfront.

**Fig 7:**
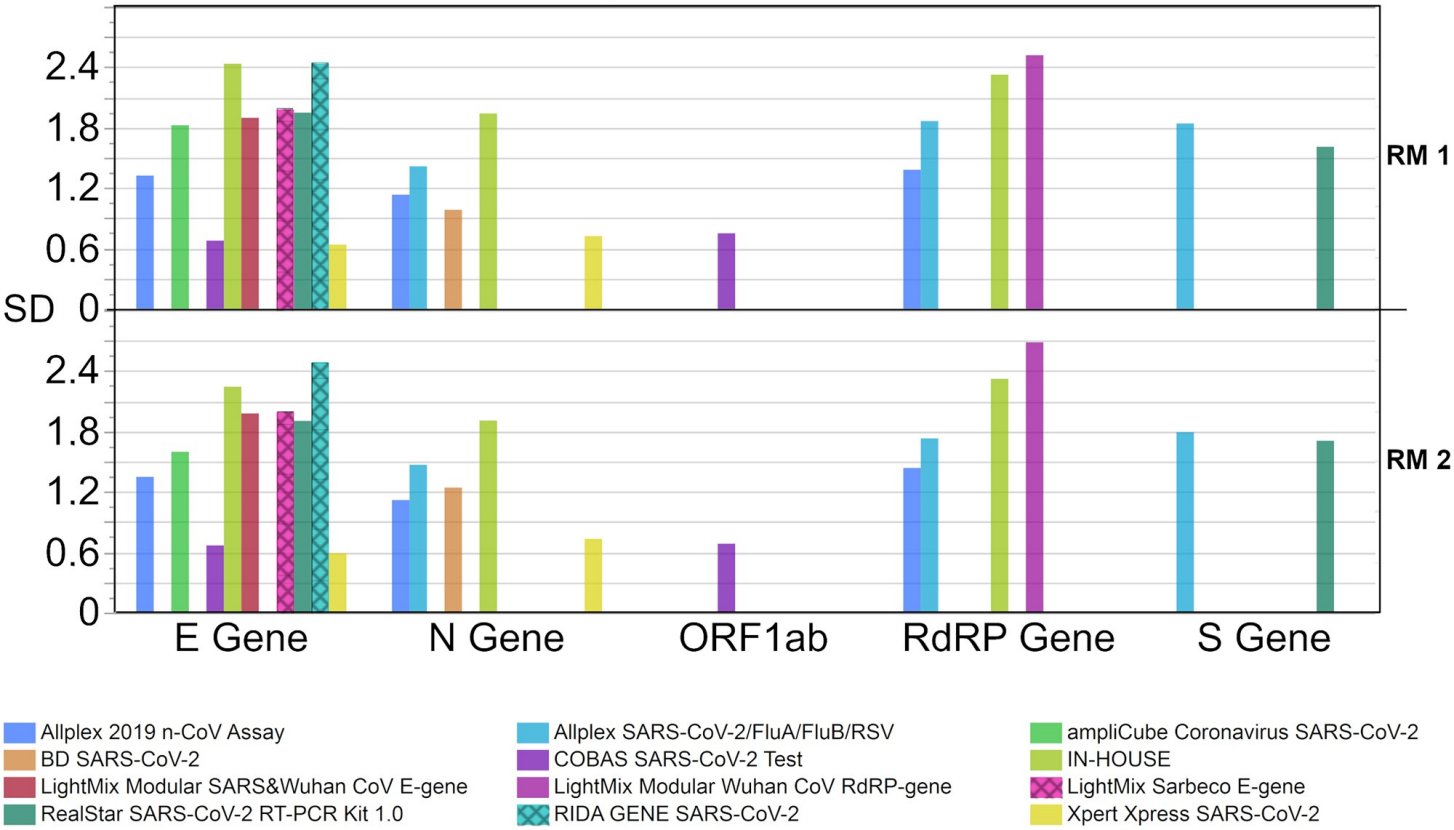
Standard deviation (SD) for the respective Ct value results per test system for the corresponding gene region. The concentration of RM 1 (blue) is estimated at roughly 10^7^ copies/mL and the concentration of RM 2 (red) is estimated at roughly 10^6^ copies/mL.

When analyzing the value distribution of single assay collectives, different test systems showed a scattering of more than 4 Ct values for their respected gene target. The lowest scatter in Ct values could be largely observed for the fully automated test systems, which include an RNA-extraction step in their procedures. These tests showed the lowest SDs in comparison to the other test systems (0.6 Ct—1.2 Ct for fully automated vs. 1.1 Ct—2.7 Ct for other systems). Interestingly, the heterogenous in-house tests had similar SDs to some commercial assays in both the E gene and the N gene (Fig 7).

Even when comparing the Ct value distribution per individual test and respective gene region, most +/- 2 SD ranges are clearly overlapped for the two concentrations with few exceptions (Table 3).

**Table 3:** Statistical analysis of Ct value distribution per gene region and test system for RM 1 and RM 2. Additional information can be found in S3 Table.

Gene Region	Test kit	RM 1				RM 2			
		N	Mean	Median	+/- 2 SD from mean	N	Mean	Median	+/- 2 SD from mean
E gene	Allplex 2019 n-CoV Assay	33	20.7	20.5	18.0–23.3	33	24.0	23.8	21.3–26.6
	ampliCube Coronavirus SARS-CoV-2	15	22.1	21.9	18.5–25.8	15	25.3	24.7	22.1–28.5
	COBAS SARS-CoV-2 Test	38	22.4	22.3	21.1–23.8	38	25.6	25.5	24.2–26.9
	IN-HOUSE	23	23.4	23.2	18.5–28.3	23	26.6	26.4	22.1–31.1
	LightMix Modular SARS and Wuhan CoV E gene	24	22.7	22.3	18.9–26.5	24	26.0	25.8	22.0–29.9
	LightMix Sarbeco E gene	22	23.3	23.0	19.3–27.3	22	26.4	26.1	22.5–30.4
	RealStar SARS-CoV-2 RT-PCR Kit 1.0	50	21.3	21.5	17.4–25.2	50	24.4	24.6	20.6–28.2
	RIDA GENE SARS-CoV-2	68	22.9	22.3	18.0–27.7	68	26.1	25.6	21.1–31.1
	Xpert Xpress SARS-CoV-2	51	21.4	21.3	20.2–22.7	51	24.5	24.4	23.4–25.7
N gene	Allplex 2019 n-CoV Assay	33	22.7	22.5	20.4–24.9	33	26.0	26.0	23.7–28.2
	Allplex SARS-CoV-2/FluA/FluB/RSV Assay	27	20.9	21.3	18.1–23.8	27	24.0	24.1	21.1–26.9
	BD SARS-CoV-2	33	20.3	20.0	18.3–22.3	33	23.5	23.3	21.0–25.9
	IN-HOUSE	25	22.8	22.4	19.0–26.7	25	26.0	25.7	22.2–29.8
	Xpert Xpress SARS-CoV-2	56	23.4	23.5	22.0–24.9	56	26.6	26.6	25.2–28.1
ORF1ab	COBAS SARS-CoV-2 Test	49	22.2	22.1	20.6–23.7	49	25.3	25.2	23.9–26.6
RdRP gene	Allplex 2019 n-CoV Assay	32	22.6	22.5	19.8–25.4	32	26.0	25.7	23.1–28.9
	Allplex SARS-CoV-2/FluA/FluB/RSV Assay	27	21.6	21.6	17.9–25.4	27	24.8	25.3	21.4–28.3
	IN-HOUSE	17	23.1	22.2	18.4–27.7	17	26.3	25.6	21.6–30.9
	LightMix Modular Wuhan CoV RdRP gene	24	26.3	26.0	21.3–31.3	24	29.8	29.0	24.4–35.1
S gene	Allplex SARS-CoV-2/FluA/FluB/RSV Assay	29	20.8	20.3	17.1–24.5	29	23.9	23.7	20.3–27.5
	RealStar SARS-CoV-2 RT-PCR Kit 1.0	49	21.4	21.3	18.2–24.6	49	24.5	24.4	21.1–27.9

**Fig 8:**
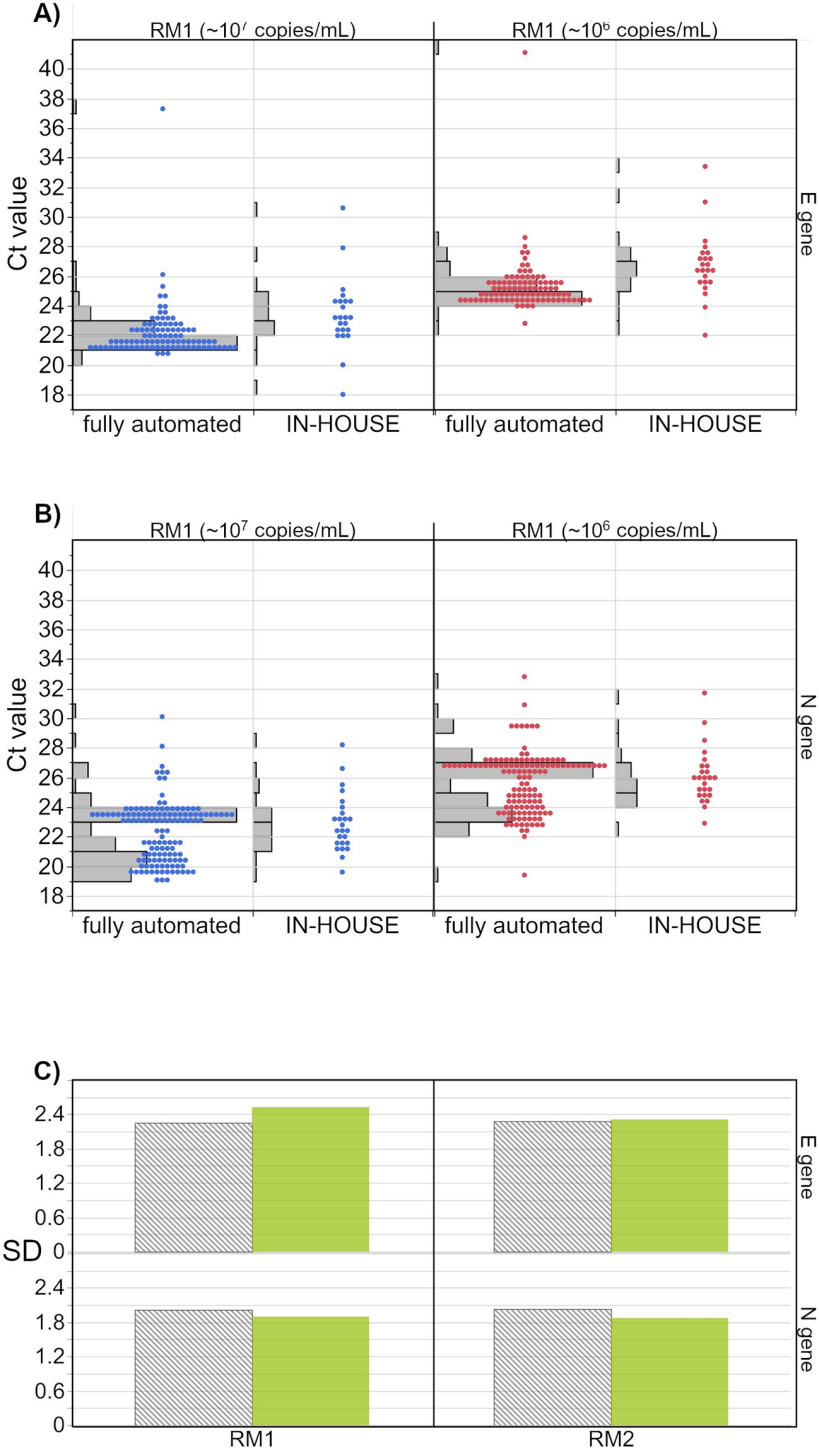
Comparison of Ct values generated by in-house tests with fully automated systems for both samples for the E gene (A), the N gene (B) as well as the corresponding standard deviations (SD). The SD were generated by combining all results obtained by the corresponding test group.

Since the ‘in-house’ collective comprises a high number of different assays and test protocols, comparability with individual assays is not very informative. For this reason, we have added a comparison of the Ct values reported for the diverse and often rather manual in-house workflows with all results for the otherwise fully automated test systems (Fig 8). Fully automated systems are those in which extraction and amplification are technically coupled and no manual step is required in between. Not included in the collective of fully automated tests were those without integrated extraction or with optional full automation due to insufficient comparability or because there was no information about how the test was conducted. A separate comparison of the results for the N gene (Fig 8B) reveals that the two groups, the fully automated tests and the in-house tests, differed only slightly in terms of median values (0.7 Ct RM 1; 0.6 Ct RM 2). For the automated systems, two clearly distinguishable collectives were apparent for both samples. In the case of the E gene (Fig 8A), the automated systems yielded lower median values than the in-house collectives (1.6 Ct RM 1; 1.7 Ct RM 2) (Table 4). The SDs were similar for in-house and automated systems for both gene regions (Fig 8C).

**Table 4:** Statistical analysis of Ct value distribution per gene region and for fully automated systems and in-house assays for RM 1 and RM 2.

		RM 1				RM 2			
Gene Region	System	N	Mean	Median	+/- 2 SD from mean	N	Mean	Median	+/- 2 SD from mean
E gene	fully automated	96	22.1	21.6	18.4–25.8	96	25.2	24.7	21.4–28.9
	in-house	23	23.4	23.2	18.4–28.5	23	26.6	26.4	22.0–31.3
N gene	fully automated	130	22.3	23.1	18.3–26.3	130	25.5	26.3	21.5–29.6
	in-house	25	22.8	22.4	18.9–26.5	25	26.0	25.7	22.1–29.6

#### 3.2.4 Performance of individual laboratories

**Fig 9:**
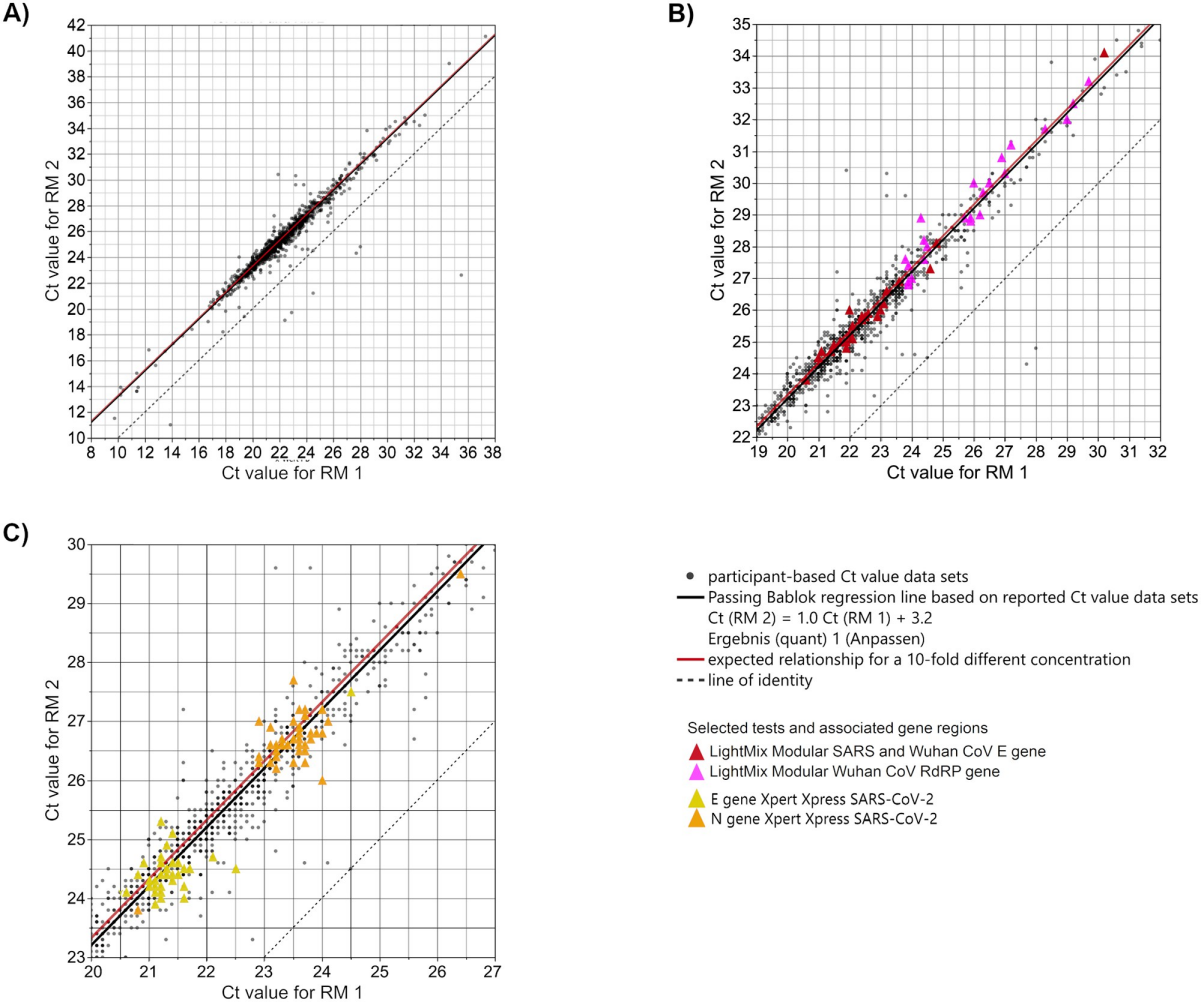
Analysis of participant-based Ct value correlation of both samples (A) with highlighted LightMix test systems (B) and Xpert Xpress test system (C). The concentration of RM 1 is estimated at roughly 10^7^ copies/mL and the concentration of RM 2 is estimated at roughly 10^6^ copies/mL. The results of all laboratories are plotted for both samples and corresponding Passing Bablok regression is shown (black regression line Ct (RM 2) = 1.0 Ct (RM 1) + 3.2), corresponding 95%-confidence limits are narrow and cannot be distinguished from the Passing Bablok regression line. Grey dashed line: line of identity. Exemplary test kits are highlighted with colour. The red line marks the expected relationship of Ct values for the two samples with concentration differences of a power of ten: Ct (RM 2) = 1.0 Ct (RM 1) + 3.32.

To check whether the laboratories could recognize the 10-fold difference of the SARS-CoV-2 viral load between RM 1 and RM 2, all results of the respectively reported Ct values were correlated. Fig 9A summarizes the Ct values obtained from all laboratories with their different test systems and different target gene regions. The Ct value difference between RM 1 (~10^7^ copies/mL) and RM 2 (~10^6^ copies/mL), representing a 10-fold concentration difference, is expected to be 3.32 cycles. A Passing Bablok fit was performed and gave a linear equation of Ct (RM 2) = 1.0 Ct (RM 1) + 3.2 (black line in Fig 9). Along the Passing Bablok regression line, the results scattered up to around 30 Ct values. However, the Passing Bablok regression line deviated only slightly from the expected Ct value relationship for the two RMs with a concentration difference of a power of ten (Ct (RM 2) = 1.0 Ct (RM 1) + 3.32). For comparison see the red line in Fig 9 (expected Ct value relationship) versus the black line (Passing Bablok regression line for observed Ct value). The y-axis interception is equal to the expected Ct value difference for both samples. Results below the regression line were provided by laboratories, of which the difference in the reported Ct values for both samples is smaller than the 3.3 Ct value.

Even when the Ct value distribution for single test collectives showed a clear overlap for the two RMs (Fig 6, Table 3) and despite the overall scatter in Ct level (up to ~ 30 Ct values), the good correlation between the SARS-CoV-2 RNA loads of RM 1 and RM 2 indicated that nearly all individual laboratories were clearly able to distinguish between both RMs based on their Ct values. This statement applies provided that for each individual laboratory the respective test used and the corresponding target gene are taken into consideration.

Fig 9B, an enlarged section of Fig 9A, shows a strong scattering of the Ct values in the lower and upper Ct value range respectively for two selected test collectives. The results of the collective that used the ‘LightMix Modular SARS and Wuhan CoV E gene Assay’ strongly scatter in the lower Ct value range, whereas the results of the collective that used the ‘LightMix Modular CoV RdRP gene Assay’ show strong scattering in the upper Ct value range.

In contrast, Fig 9C, another enlarged section of Fig 9A, shows that for another test, the ‘Xpert Xpress SARS-CoV-2 Assay’, the Ct values corresponding to the E and N gene, respectively, only scatter slightly. The respective Ct values, however, are easily discernible from one another.

In addition, the Ct value differences between the two RMs were calculated for each laboratory data set. Looking at the distribution of the Ct value differences for individual assays, the median Ct value differences were very similar, ranging in value from 3.1 to 3.4 Ct (Table 5).

**Table 5:** Statistical analysis of Ct value differences per gene region and test system for RM 1 and RM 2: Mean, median and 95%-CI of mean.

Gene Region	Test kit	N	Mean	Median	95%-CI of mean
E gene	Allplex 2019 n-CoV Assay	33	3.3	3.3	2.6–3.9
	ampliCube Coronavirus SARS-CoV-2	15	3.2	3.3	1.8–4.5
	COBAS SARS-CoV-2 Test	38	3.1	3.1	2.8–3.4
	IN-HOUSE	23	3.2	3.2	1.8–4.7
	LightMix Modular SARS and Wuhan CoV E gene	24	3.3	3.3	2.1–4.4
	LightMix Sarbeco E gene	22	3.1	3.2	1.9–4.4
	RealStar SARS-CoV-2 RT-PCR Kit 1.0	50	3.1	3.1	2.4–3.9
	RIDA GENE SARS-CoV-2	68	3.3	3.2	2.4–4.1
	Xpert Xpress SARS-CoV-2	51	3.1	3.1	2.9–3.4
N gene	Allplex 2019 n-CoV Assay	33	3.3	3.3	2.7–3.9
	Allplex SARS-CoV-2/FluA/FluB/RSV Assay	27	3.1	3.1	2.3–3.9
	BD SARS-CoV-2	33	3.2	3.2	2.6–3.7
	IN-HOUSE	25	3.1	3.2	2.0–4.3
	Xpert Xpress SARS-CoV-2	56	3.2	3.2	2.9–3.5
ORF1ab	COBAS SARS-CoV-2 Test	49	3.1	3.1	2.8–3.4
RdRP gene	Allplex 2019 n-CoV Assay	32	3.4	3.4	2.7–4.1
	Allplex SARS-CoV-2/FluA/FluB/RSV Assay	27	3.2	3.3	2.2–4.2
	IN-HOUSE	17	3.2	3.2	1.5–4.9
	LightMix Modular Wuhan CoV RdRP gene	24	3.5	3.4	1.9–5.0
S gene	Allplex SARS-CoV-2/FluA/FluB/RSV Assay	29	3.1	3.2	2.1–4.1
	RealStar SARS-CoV-2 RT-PCR Kit 1.0	49	3.1	3.1	2.4–3.7

**Fig 10:**
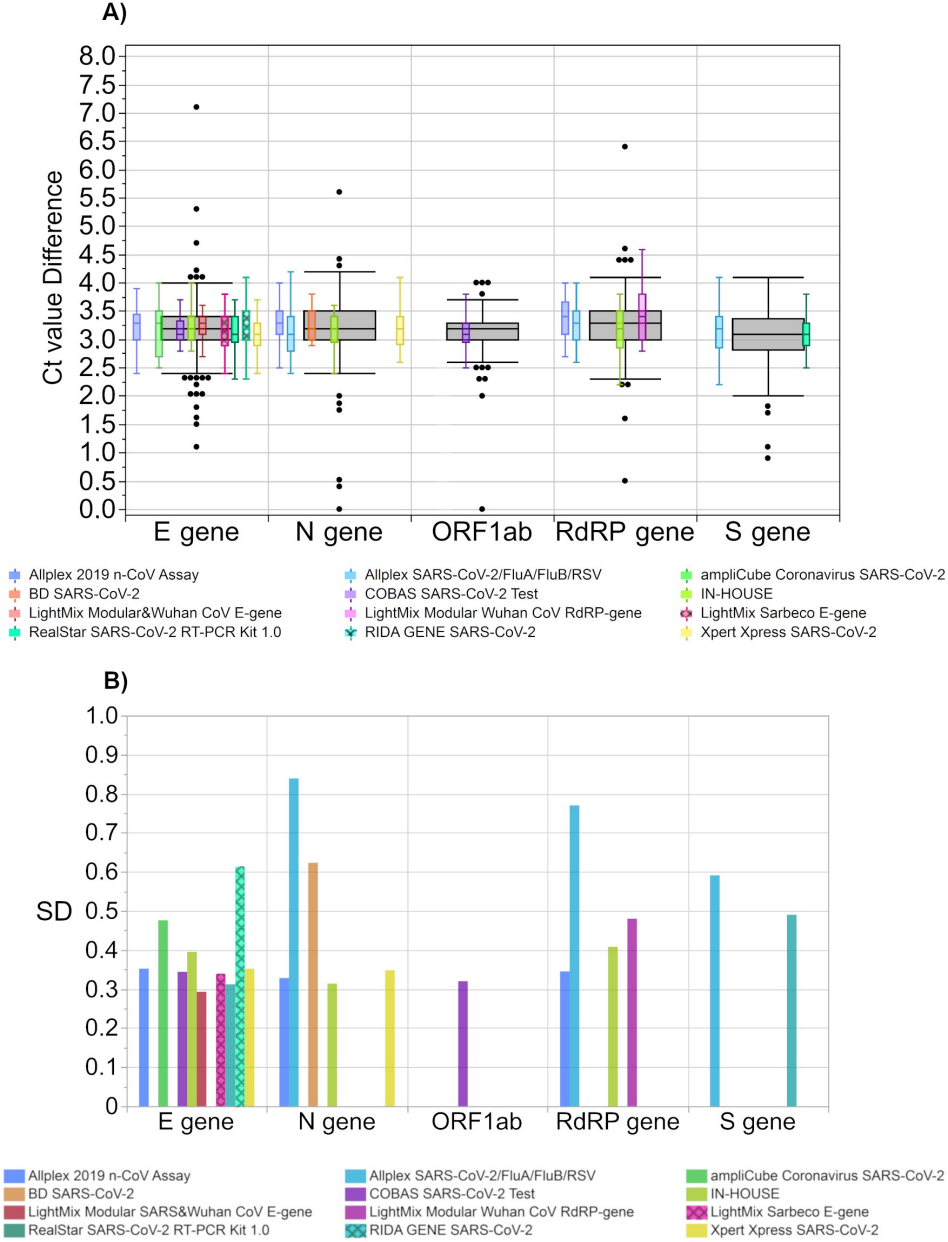
(A) Ct value difference between both samples per test system and (B) their respective standard deviations (SD). The grey boxes display all results for the respective sample, and the distributions of specific manufacturer-based collectives are illustrated as smaller, colored box plots in overlay with the total results. Outlier were excluded from colored boxes. For all boxes, the whiskers stretch from the 1st quartile—1.5*(interquartile range) to the 3rd quartile + 1.5*(interquartile range).

Nevertheless, some uncertainty remains as the differences between the Ct values within the test collectives scattered up over a 1.5 Ct value (Fig 10A). The SDs for the Ct value differences ranged from a minimum of 0.293 Ct for the ‘LightMix Modular SARS and Wuhan CoV E gene’ up to 0.839 Ct for the ‘Allplex SARS-CoV-2/FluA/FluB/RSV Assay’ (N gene) (Fig 10B).

## 4 Discussion

Due to the acute demand for SARS-CoV-2 testing and a rapid adaptation of the offers on the market, there is a high range of assay types and analytical approaches [29]. Major differences consist of manual tests (commercial or in-house tests) or automated assay procedures (high throughput or single unit tests), target genes, extraction and amplification procedures (separate or combined platforms) or even extraction-free assays [30–35]. The multitude in analytical approaches and the fact that no International Standard or RM existed in the first months of the pandemic are enough to suggest a lack of homogeneity in the results reported by the laboratories. Many articles on assay evaluations or method comparisons have been published up to now, reporting a partially wide scatter in Ct values, especially for low SARS-CoV-2 viral load [7–11].

The EQA scheme for SARS-CoV-2 genome detection performed by INSTAND as early as April 2020 showed that the participating laboratories were able to easily detect the virus for well-characterized SARS-CoV-2 quality control samples. However, the Ct values reported for the same EQA sample varied considerably depending on the individual laboratory, the test system, and the target gene [13,14]. The same observation was reported by other external quality assessment schemes [8–19,36,37].

The Ct values of SARS-CoV-2 RT-qPCR results were already used at the beginning of the pandemic to estimate the SARS-CoV-2 viral load in diagnostic specimens such as nasopharyngeal swabs [2,12,16,17,38–41], especially in the context of making clinical decisions surrounding COVID-19 patients (e.g. discharge of patients from isolation). These decisions were made despite knowing that SARS-CoV-2 genome detection essentially depends not only on the quality of the collected test material but also on the genome detection method used by the laboratory [8–10,36,37] and that detection of SARS-CoV-2-RNA does not proof infectivity of the sample.

In this study we analyzed a total of 1,097 data sets for the two reference materials RM 1 and RM 2, which were designed to determine whether an anchoring between the obtained Ct values and the specified SARS-CoV-2 viral load could be achieved.

First, sample stability under ‘real-life laboratory conditions’ was confirmed through horizontal linear regression of all results over the timeframe of the study. This indicates that this lyophilized RM shows a suitable storage stability of at least five months (Fig 3).

A pre-evaluation of the results from the first shipment already showed that across laboratories the concentrations of ~10^6^ and ~10^7^ copies/mL cannot be distinguished in all Ct values since there was a clear overlap in value distribution [42]. Such dispersion of Ct values, also observed in this study, might arise from the fact that different SARS-CoV-2 NAT protocols target different gene regions [43]. The most common SARS-CoV-2 target gene in RT-qPCR analysis is the envelope (E) gene, detected by eight of the eleven different assays analyzed in this paper as well as many in-house protocols. The nucleocapsid (N) gene and RNA-dependent RNA polymerase (RdRP) gene are targeted by four of the major test manufacturers and by in-house test protocols. To analyze how the different gene regions contribute to this strong scattering of results, we stratified the reported values by gene region and RM. Even though the median values of the different samples were clearly distinguishable for each gene region, the distribution of the reported Ct values showed a strong overlap (Fig 4). This clearly indicates that it is not possible to define a universal Ct value to estimate the viral load on the basis of a selected gene region, since apparently different assays provide markedly variable differences in Ct for different genes.

A further stratification of the data set for individual assays shows that only a few tests were able to distinguish between both SARS-CoV-2 concentrations with a 95% certainty. Furthermore, some gene targets showed a higher dispersion of the median values of the different test systems. For N gene and the RdRP gene, the overall dispersion of the Ct values was slightly higher than for the E gene. The assay-dependent medians of the Ct values differed in about 3 Ct values for the N gene and 4 Ct values for the RdRP gene (Table 2). There are several factors that might be responsible for the differences in value output between the different test systems and their respectively targeted gene regions. For example, the stringency in the binding of the various assay-specific primers might be the cause of the different Ct value levels. In the case of the N gene, differences in test-dependent Ct values might be further influenced by targeting two basically different sites: N1 and N2 [44]. The smallest gene-dependent overall dispersion of Ct values was observed for the ORF1ab (Fig 6). Here, the smaller number of compatible tests and the lower analytical diversity might contribute to the lesser observed dispersion. Another reason for the observed dispersion of median Ct values of different test systems may be due to the fact that prior to the regular PCR reaction some tests use pre-amplification cycles that are not included in the output Ct value (e.g. Abbott RealTime SARS-CoV-2 and Seegene Allplex SARS-CoV-2/FluA/FluB/RSV Assay, respectively).

It can further be observed that the Ct value distribution is lower in assays where RNA extraction is part of an automated test system, such as the ‘COBAS SARS-CoV-2 Test’. This is also true for the single-unit test, ‘Xpert Xpress SARS-CoV-2’, which is a closed system. However, assays representing open platforms, such as ‘Allplex 2019-nCoV Assay’ and ‘RealStar SARS-CoV-2 RT-PCR Kit 1.0’ showed an increased scattering of the reported Ct values (Table 3). The reason for this might be that these open platforms are often used in combination with different extraction procedures. As for open platforms, extraction procedures other than those recommended by the manufacturer for their PCR system are sometimes used by the laboratories, further increasing the multitude of different extraction kits and PCR combinations in addition to different protocols, which in turn further amplifies the diversity in the analytical approaches. Despite the large amount of available data for this study, no statement can be made about the impact of extraction protocols on the reported Ct values as the individual workflows varied too much. Systematic studies with comparable and sufficiently large collectives would be needed to further address this question.

When comparing the Ct value distribution of manual and fully automated test systems, performance of in-house tests was found to be almost as good as that of the fully automated systems (Fig 8A and 8B). This observation is consistent with the results from Matheeussen et al. who reported an equal or even better performance of in-house tests in comparison to commercial ones [36]. In this study, the SDs for Ct values of in-house tests were between 1.9 Ct and 2.5 Ct for the two samples in comparison to the SD of all fully automated systems combined with 2.0 Ct to 2.3 Ct (Fig 8C). This pointed to a satisfyingly high degree of precision in the manual analyses. Nevertheless, the good results of the heterogenous in-house collective showed that differences in manual approaches do not have as high of an impact on the general interlaboratory comparability as INSTAND previously found for in vitro allergy diagnostic immunoassays [45,46].

A positive observation of this study was that, despite the strong overall dispersion of the assay collectives, nearly all individual laboratories were able to differentiate between the SARS-CoV-2 RNA loads of ~10^7^ copies/mL in RM 1 and ~10^6^ copies/mL in RM 2 (Figs 9 and 10) regardless of the assay used.

Although the data sets for RM 1 and RM 2 (represented by individual data points in Fig 9A) scattered considerably up to 30 Ct values along the Passing Bablok regression line, the correlation analysis of all the submitted Ct value data sets yielded a Passing Bablok regression line almost congruent with the expected relationship of Ct values for both RMs with a 10-fold concentration difference.

This argues for a successful performance of the laboratories as well as a valid functionality of the respective test systems by applying RM 1 and RM 2. This also underscores the fact that reference materials, such as those described here, as well as International Standards, such as the newly established WHO International Standard for SARS-CoV-2 RNA [47], are indispensable for the assessment of SARS-CoV-2 viral load in patient materials.

A detailed look at two test collectives highlighted in Fig 9B and 9C reveal different degrees of scattering for different test systems along the regression line. Disregarding outliers within the two different test collectives that used the LightMix systems (‘LightMix Modular SARS and Wuhan CoV E gene Assay’ and ‘LightMix Modular CoV RdRP gene’), the reported Ct value sets were scattered by about 3 Ct values for the E gene and 5 Ct values for the RdRP gene. The reason for this deviation could be that the LightMix system is modularly composed of different components, quasi as an open in-house test. In contrast, the closed single unit test, the ‘Xpert Xpress SARS-CoV-2’ assay revealed two clearly separated sub-collectives for the two different gene targets, E gene and N gene (Fig 9C).

The Ct value difference for the samples with a 10-fold concentration difference is expected to be 3.32 cycles, equal to the y-axis interception of the expected relationship in Fig 9A. Looking at Ct value differences for selected test systems in Fig 10A and Table 5, all median values of the test-dependent Ct value differences were between 3.1 and 3.4 Ct values and were therefore very close to the target value of 3.32. Moreover, the scattering of the Ct value differences within individual test collectives ranged from slightly below 1 to up to 2 Ct values.

Our study confirms that the laboratories’ performance with regard to their individual SARS-CoV-2 RT-qPCR tests is good overall. It must be emphasized that a correlation between viral load and measured Ct value can only be established by anchoring the Ct values obtained with assigned viral RNA loads of suitable reference materials, such as RM 1 and RM 2 and that the Ct value is unique to the laboratory and method being used. The special feature of RM 1 and RM 2 is that their SARS-CoV-2 RNA loads had been well quantified by two different approaches, by RT-qPCR using synthetic RNA molecules (Fig 1) and by digital PCR (Fig 2). Using RM 1 and RM 2, laboratories were able to determine what a given Ct value corresponded to for their respective test method used. It is clear that a Ct value obtained by a specific test system with the corresponding target gene does not apply to other tests and other target genes. This means that, for the introduction of a new test method, such a quantitative assessment must be performed again with the reference materials. Therefore, in the case of routine diagnosis, each laboratory must define its own threshold range of SARS-CoV-2 viral load between ~10^7^ copies/mL and ~10^6^ copies/mL for each assay as well as for each target gene. RM 1 and RM 2 allow laboratory professionals to correlate their procedure-dependent Ct values to the quantitative target values to support clinical decisions, like discharging patients from isolation [12,15–19]. Quantitative statements will be increasingly needed in the future to understand the severity of a positive PCR result in immunocompromised patients with persistent viral shedding [48,49] or treatment decisions [50] once antiviral therapy is universally established. Of course, a valid clinical decision presupposes that the preanalytical phase was correctly performed when the diagnostic test sample, e.g. the nasopharyngeal swab, was collected and that reliable anamnestic information is available. In addition, the time of sample collection must be considered with respect to the time of infection.

In terms of the conclusions that can be drawn for this study, it should be noted that influencing factors such as matrix differences between the reference materials and routine diagnostic samples (commutability) as well as the presence of different virus variants were not a subject of this investigation. Further studies are necessary to gain insight into the contribution of sequence differences observed for virus variants including variants of concern (VOCs) for anchoring Ct values to viral RNA loads.

In this respect, RM 1 and RM 2 described here and future RMs are useful as candidates of a measurement standard since the quantification methods used could allow to establish traceability by direct counting of genetic copies [51]. Those would complement the implementation of the WHO International Standard for SARS-CoV-2 RNA [47,52] that is designed as international conventional calibrator, and as such defines its own international units. An advantage of adjusted reference materials is the ability to react quickly to the state of the pandemic, since new VOCs have emerged and may still emerge during the pandemic [53,54].

## 5 Conclusion

In summary, this study demonstrates that the participating laboratories were proficient with regard to their applied tests for genome detection of SARS-CoV-2 in detecting the 10-fold concentration difference between RM 1 (~10^7^ copies/mL) and RM 2 (~10^6^ copies/mL) and thereby in anchoring their obtained Ct values with the assigned SARS-CoV-2 RNA loads of RM 1 and RM 2, respectively.

However, our study clearly shows that it is not possible to define a universal Ct value related to a given SARS-CoV-2 RNA load. Therefore, for clinical guidance based on SARS-CoV-2 viral loads, such as in the context of discharge management, Ct values should not be used as the sole measure. It is imperative that each individual laboratory uses its individual test system to link the specified RNA viral loads of reference materials such as RM 1 and RM 2 to the corresponding Ct values for the respective gene region.

As additionally, Ct values can vary widely between different runs on the same instrument, it is recommended that RMs described here or similar control materials should be applied as defined run controls to anchor Ct values with copy-based units such as genome copies or international units to monitor the stability of the test system applied.

Following the track of our study, using reference materials for quantifying SARS-CoV-2 RNA in patient specimens should pave the way for a harmonization of results from various test systems for the detection of SARS-CoV-2 RNA.

## Supporting information

S1 Fig Depiction of the analyses by the 4 laboratories (mentioned in Section 2.4) of their genome detection of SARS-CoV-2 for RM 1 and RM 2. Each data point (Ct value) corresponds to a single measurement result. The following data are shown for the Ct values of RM 1 and RM 2: mean (solid vertical line); prediction interval (95% probability; dotted vertical line) for RM 1 (denoted by *) and RM 2 (denoted by **). Prediction intervals for the difference in Ct values (horizontal line with arrows) of RM 1 and RM 2 are marked with ***. Measurements A) and B) were done by laboratory 1, measurements C) and D) were provided by laboratory 2, measurement E) was done by laboratory 3 and F) by laboratory 4.
(TIFF)

S2 Fig Development of Ct values for both reference samples over time for N gene results (A), for ORF1ab results (B), for RdRP gene results (C) and for S gene results (D). Each symbol represents one measurement. Dots are participant results and triangles are results from the sample provider.
(TIFF)

S1 Table Quantitative pre-characterization of SARS-CoV-2 in the cell culture supernatant—Applied volumes for digital PCR measurements by the three National Metrology Institutes, NML, NIST and PTB, for quantification of the INSTAND EQA sample 340066 of the INSTAND EQA scheme (340) Virus Genome Detection Coronaviruses incl. SARS-CoV-2 June/July 2020.
(DOCX)

S2 Table Quantitative pre-characterization of SARS-CoV-2 in the cell culture supernatant—Measurement results determined by digital PCR by the three National Metrology Institutes, NML, NIST and PTB, for sample 340066 of INSTAND EQA scheme (340) Virus Genome Detection Coronaviruses incl. SARS-CoV-2 June/July 2020.
(DOCX)

S3 Table Applied volumes for digital PCR measurements by the three National Metrology Institutes, NML, NIST and PTB, for quantification of RM 1 and RM 2. (DOCX)

S4 Table Measurement results determined by digital PCR by NML, NIST and PTB for RM 1 and RM 2. (DOCX)

S1 Data Measurement results of the 4 laboratories (mentioned in Section 2.4) of their genome detection of SARS-CoV-2 for RM 1 and RM 2. (XLSX)

S2 Data Participant results for both reference materials and all three shipments. This table contains the raw results of the EQA participants without any correction. Results that were excluded from evaluation, e.g. due to being most likely sample swaps, are highlighted in orange. The results from the supplier were only included in the time-dependent analysis and were excluded from the main analysis.
(XLSX)

S3 Data Basic statistics for both reference materials per gene region, and per assay and gene region. (XLSX)
