# Supplementary figures and images for: RNA reference materials with defined viral RNA loads of SARS-CoV-2—A useful tool towards a better PCR assay harmonization
Source: PLoS One. 2022 Jan 20;17(1):e0262656. doi: 10.1371/journal.pone.0262656 (PMC8775330; doi:10.1371/journal.pone.0262656)

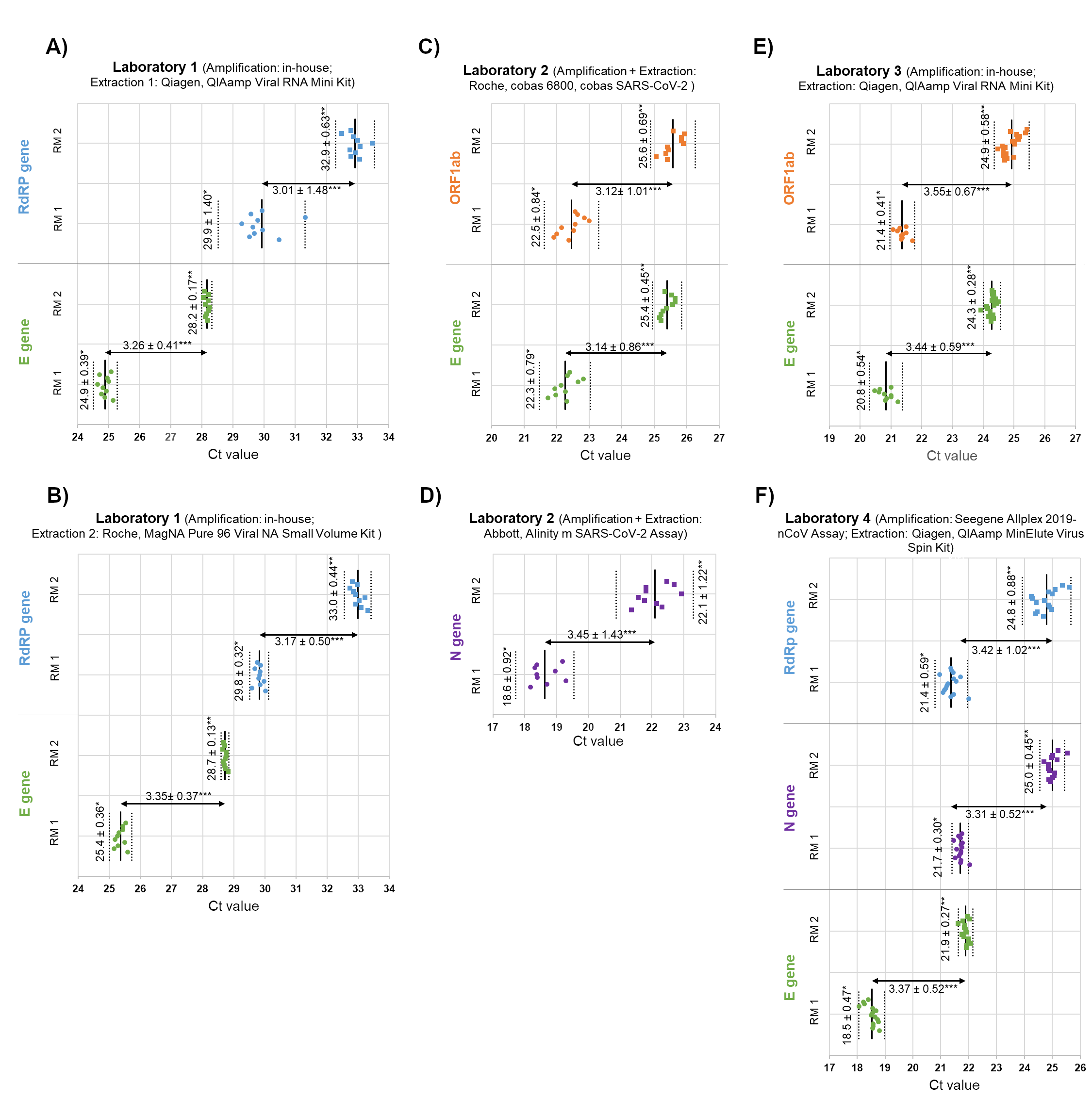

Supplement: S1 Fig — Each data point (Ct value) corresponds to a single measurement result. The following data are shown for the Ct values of RM 1 and RM 2: mean (solid vertical line); prediction interval (95% probability; dotted vertical line) for RM 1 (denoted by *) and RM 2 (denoted by **). Prediction intervals for the difference in Ct values (horizontal line with arrows) of RM 1 and RM 2 are marked with ***. Measurements A) and B) were done by laboratory 1, measurements C) and D) were provided by laboratory 2, measurement E) was done by laboratory 3 and F) by laboratory 4. (TIFF) [file pone.0262656.s001.tiff]

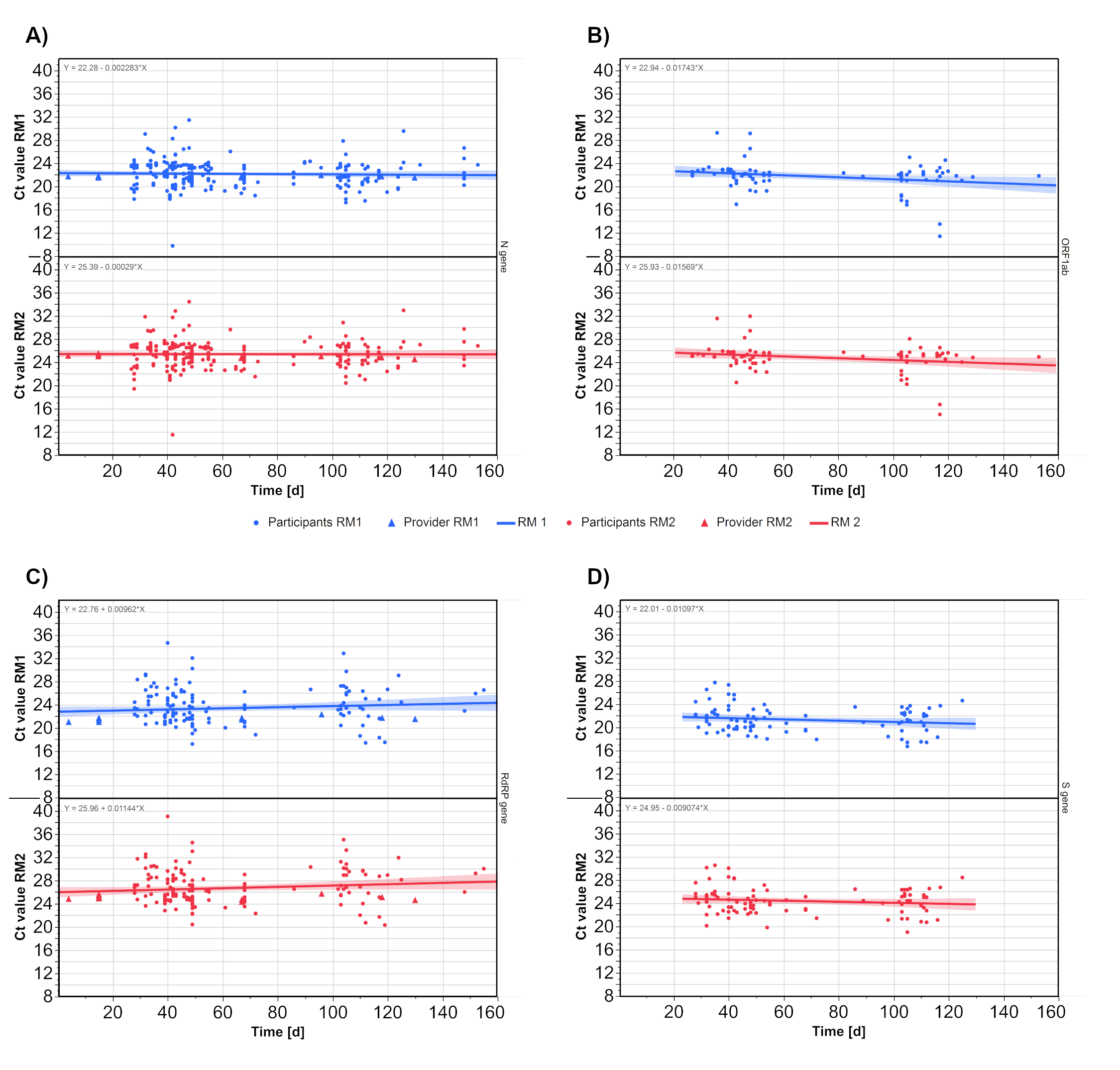

Supplement: S2 Fig — Each symbol represents one measurement. Dots are participant results and triangles are results from the sample provider. (TIFF) [file pone.0262656.s002.tiff]
