# Supplementary material for: RNA reference materials with defined viral RNA loads of SARS-CoV-2—A useful tool towards a better PCR assay harmonization
Source: PLoS One. 2022 Jan 20;17(1):e0262656. doi: 10.1371/journal.pone.0262656 (PMC8775330; doi:10.1371/journal.pone.0262656)
Supplement: S1 Table — SARS-CoV-2 June/July 2020. (DOCX) [file pone.0262656.s003.docx]

**S1 Table.** **Quantitative pre-characterization of SARS-CoV-2 in the cell culture supernatant - applied volumes for digital PCR measurements by the three National Metrology Institutes, NML, NIST and PTB, for quantification of the INSTAND EQA sample 340066 of the INSTAND EQA scheme (340) Virus Genome Detection Coronaviruses incl. SARS-CoV-2 June/July 2020.**

| Laboratory | Partition volume (nL) | Template volume (μL) | Reaction volume (μL) | Extraction input (μL) | Elution volume (μL) |
| --- | --- | --- | --- | --- | --- |
| NML | 0.7760 | 7 | 20 | 140 | 60 |
| NIST | 0.7472 | 10 | 22 | 140 | 60 |
| PTB | 0.8500 | 8 | 20 | 200 | 62 |
