# Supplementary material for: RNA reference materials with defined viral RNA loads of SARS-CoV-2—A useful tool towards a better PCR assay harmonization
Source: PLoS One. 2022 Jan 20;17(1):e0262656. doi: 10.1371/journal.pone.0262656 (PMC8775330; doi:10.1371/journal.pone.0262656)
Supplement: S2 Table — SARS-CoV-2 June/July 2020. (DOCX) [file pone.0262656.s004.docx]

**S2 Table.** **Quantitative pre-characterization of SARS-CoV-2 in the cell culture supernatant - measurement results determined by digital PCR by the three National Metrology Institutes, NML, NIST and PTB, for sample 340066 of INSTAND EQA scheme (340) Virus Genome Detection Coronaviruses incl. SARS-CoV-2 June/July 2020.**

| **Laboratory** | **Assay** | **Vial** | **Number of measurement** | **SARS-CoV-2 viral load copies/mL** |
| --- | --- | --- | --- | --- |
| NML | CDC N2 | 1 | 1 | 2124 |
|  |  |  | 2 | 1365 |
|  |  |  | 3 | 700 |
|  |  | 2 | 1 | 2079 |
|  |  |  | 2 | 1625 |
|  |  |  | 3 | 980 |
|  |  | 3 | 1 | 1736 |
|  |  |  | 2 | 1401 |
|  |  |  | 3 | 777 |
| PTB | China N | 1 | 1 | 962 |
|  |  |  | 2 | 1055 |
|  |  |  | 3 | 855 |
|  |  | 2 | 1 | 784 |
|  |  |  | 2 | 671 |
|  |  |  | 3 | 1432 |
|  |  | 3 | 1 | 2246 |
|  |  |  | 2 | 2050 |
|  |  |  | 3 | 1939 |
| NIST | China N | 1 | 1 | 1580 |
|  |  |  | 2 | 1276 |
|  |  |  | 3 | 1243 |
|  |  |  | 4 | 1317 |
|  | CDC N1 |  | 1 | 1739 |
|  |  |  | 2 | 649 |
|  |  |  | 3 | 1665 |
|  |  |  | 4 | 1391 |
|  | CDC N2 |  | 1 | 1443 |
|  |  |  | 2 | 2105 |
|  |  |  | 3 | 2082 |
|  |  |  | 4 | 1808 |
|  | China N | 2 | 1 | 2324 |
|  |  |  | 2 | 991 |
|  |  |  | 3 | 1692 |
|  |  |  | 4 | 1323 |
|  |  |  | 5 | 1832 |
|  |  |  | 6 | 1275 |
|  | CDC N1 |  | 1 | 1255 |
|  |  |  | 2 | 924 |
|  |  |  | 3 | 1719 |
|  |  |  | 4 | 2004 |
|  |  |  | 5 | 1372 |
|  |  |  | 6 | 972 |
|  | CDC N2 |  | 1 | 1958 |
|  |  |  | 2 | 1807 |
|  |  |  | 3 | 1624 |
|  |  |  | 4 | 2365 |
|  |  |  | 5 | 1794 |
|  |  |  | 6 | 1502 |
|  | China N | 3 | 1 | 1608 |
|  |  |  | 2 | 1742 |
|  |  |  | 3 | 1254 |
|  |  |  | 4 | 1342 |
|  |  |  | 5 | 1887 |
|  |  |  | 6 | 2066 |
|  | CDC N1 |  | 1 | 1165 |
|  |  |  | 2 | 1401 |
|  |  |  | 3 | 1611 |
|  |  |  | 4 | 1597 |
|  |  |  | 5 | 1772 |
|  |  |  | 6 | 1756 |
|  | CDC N2 |  | 1 | 1956 |
|  |  |  | 2 | 1926 |
|  |  |  | 3 | 2030 |
|  |  |  | 4 | 2334 |
|  |  |  | 5 | 1897 |
|  |  |  | 6 | 2027 |
