# Supplementary material for: RNA reference materials with defined viral RNA loads of SARS-CoV-2—A useful tool towards a better PCR assay harmonization
Source: PLoS One. 2022 Jan 20;17(1):e0262656. doi: 10.1371/journal.pone.0262656 (PMC8775330; doi:10.1371/journal.pone.0262656)
Supplement: S3 Table — (DOCX) [file pone.0262656.s005.docx]

**S3 Table.** **Applied volumes for digital PCR measurements by the three National Metrology Institutes, NML, NIST and PTB, for quantification of RM 1 and RM 2.**

| Laboratory | Partition volume (nL) | Template volume (μL) | Reaction volume (μL) | Extraction input (μL) | Elution volume (μL) |
| --- | --- | --- | --- | --- | --- |
| NML | 0.7760 | 5 | 20 | 140 | 60 |
| NIST | 0.7472 | 10 | 22 | 140 | 60 |
| PTB | 0.8500 | 8 | 20 | 200 | 62 |
