# Supplementary material for: RNA reference materials with defined viral RNA loads of SARS-CoV-2—A useful tool towards a better PCR assay harmonization
Source: PLoS One. 2022 Jan 20;17(1):e0262656. doi: 10.1371/journal.pone.0262656 (PMC8775330; doi:10.1371/journal.pone.0262656)
Supplement: S4 Table — (DOCX) [file pone.0262656.s006.docx]

**S4 Table. Measurement results determined by digital PCR by NML, NIST and PTB for RM 1 and RM 2.**

**RM 1:**

| **Laboratory** | **Assay** | **Vial** | **Number of measurement** | **SARS-CoV-2 viral load copies/mL** |
| --- | --- | --- | --- | --- |
| NML | CDC N2 | 1 | 1 | 13205639 |
|  |  |  | 2 | 13290891 |
|  |  |  | 3 | 13631137 |
|  |  | 2 | 1 | 12603288 |
|  |  |  | 2 | 11345956 |
|  |  |  | 3 | 13009660 |
|  |  | 3 | 1 | 13060023 |
|  |  |  | 2 | 12628266 |
|  |  |  | 3 | 13498702 |
| PTB | China N | 1 | 1 | 11157095 |
|  |  |  | 2 | 11416916 |
|  |  |  | 3 | 11357263 |
|  |  |  | 4 | 10532057 |
|  |  | 2 | 1 | 10285071 |
|  |  |  | 2 | 10112953 |
|  |  |  | 3 | 10669946 |
|  |  |  | 4 | 10624410 |
|  |  |  | 5 | 10829851 |
|  |  |  | 6 | 10847947 |
|  |  |  | 7 | 11010298 |
|  |  |  | 8 | 10194718 |
|  |  | 3 | 1 | 10746427 |
|  |  |  | 2 | 10765618 |
|  |  |  | 3 | 10681383 |
|  |  |  | 4 | 10856263 |
| NIST | CDC N2 | 1 | 1 | 12030189 |
|  |  |  | 2 | 12162630 |
|  |  |  | 3 | 11934701 |
|  |  |  | 4 | 11753484 |
|  |  | 2 | 1 | 14160167 |
|  |  |  | 2 | 15305685 |
|  |  |  | 3 | 14806695 |
|  |  |  | 4 | 14949943 |
|  |  | 3 | 1 | 13706729 |
|  |  |  | 2 | 13684620 |
|  |  |  | 3 | 13805143 |
|  |  |  | 4 | 13713558 |

**RM 2:**

| **Laboratory** | **Assay** | **Vial** | **Number of measurement** | **SARS-CoV-2 viral load copies/mL** |
| --- | --- | --- | --- | --- |
| NML | CDC N2 | 1 | 1 | 1370963 |
|  |  |  | 2 | 1272497 |
|  |  |  | 3 | 1265123 |
|  |  | 2 | 1 | 1309098 |
|  |  |  | 2 | 1262168 |
|  |  |  | 3 | 1334721 |
|  |  | 3 | 1 | 1407261 |
|  |  |  | 2 | 1439154 |
|  |  |  | 3 | 1327157 |
| PTB | China N | 1 | 1 | 1132378 |
|  |  |  | 2 | 1066345 |
|  |  |  | 3 | 1110716 |
|  |  | 2 | 1 | 1031002 |
|  |  |  | 2 | 1082329 |
|  |  |  | 3 | 1093688 |
|  |  |  | 4 | 1101992 |
|  |  |  | 5 | 1041248 |
|  |  |  | 6 | 1145245 |
|  |  | 3 | 1 | 1037909 |
|  |  |  | 2 | 1037154 |
|  |  |  | 3 | 1079932 |
|  |  |  | 4 | 1031725 |
|  |  |  | 5 | 1089074 |
|  |  |  | 6 | 1058688 |
|  |  |  | 7 | 1051934 |
|  |  |  | 8 | 1025429 |
| NIST | CDC N2 | 1 | 1 | 1154658 |
|  |  |  | 2 | 1128666 |
|  |  |  | 3 | 1100124 |
|  |  |  | 4 | 1137373 |
|  |  | 2 | 1 | 1187193 |
|  |  |  | 2 | 1259983 |
|  |  |  | 3 | 1296443 |
|  |  |  | 4 | 1290441 |
|  |  | 3 | 1 | 1418613 |
|  |  |  | 2 | 1458520 |
|  |  |  | 3 | 1416160 |
|  |  |  | 4 | 1406721 |
